# Supplementary material for: SETBP1 accumulation induces P53 inhibition and genotoxic stress in neural progenitors underlying neurodegeneration in Schinzel-Giedion syndrome
Source: Nat Commun. 2021 Jun 30;12:4050. doi: 10.1038/s41467-021-24391-3 (PMC8245514; doi:10.1038/s41467-021-24391-3)
Supplement: Supplementary file 1 — Supplementary Information [file 41467_2021_24391_MOESM1_ESM.pdf]

Supplementary Figure 1

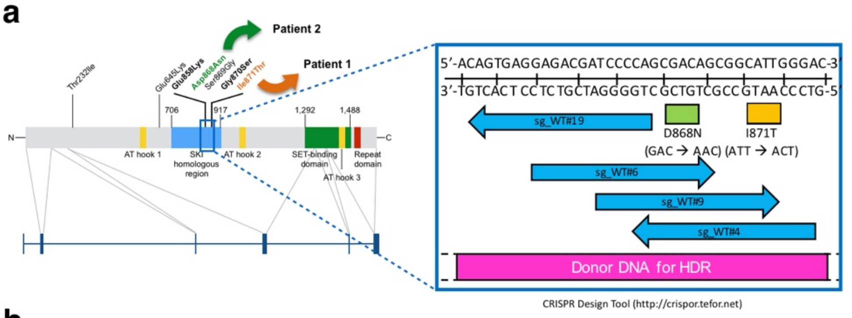

sgI871T_9 OFF TARGETS

Gene	Chr	Sequence	Mismatches	Score
CDK5	7	5'-ACCCCAGCCTCAGCAGCACTTGG-3'	4	0.45
C1S	12	5'-CCCCCAGGGACAGCAGCACAGGG-3'	4	0.29
EPHB3	3	5'-TCCCCACTCACGGCGGCACTGGG-3'	4	0.27
HGS/RP13	17	5'-CCGCCCGCGACGGCGGCACTGGG-3'	4	0.14
DENND6B	22	5'-TCCCCAGTGGTGGCGGCACTGGG-3'	4	0.11

sgD868N_6 OFF TARGETS

Gene	Chr	Sequence	Mismatches	Score
BSG	19	5'-GAGTCAGTCCCCAGCAAAAGTGG-3'	4	0.24
DYNC1H1	14	5'-GAGACAGTCCCCAGCAATGGTGG-3'	4	0.17
CDHR4	3	5'-GAGACGGTCCACAGCAAAGGTGG-3'	4	0.11
DLK1	14	5'-GTGAGGAACACCAGCAACAGGAG-3'	4	0.08
HAUS6	9	5'-GAGACTAGCCTCAGCAACACAGG-3'	4	0.06

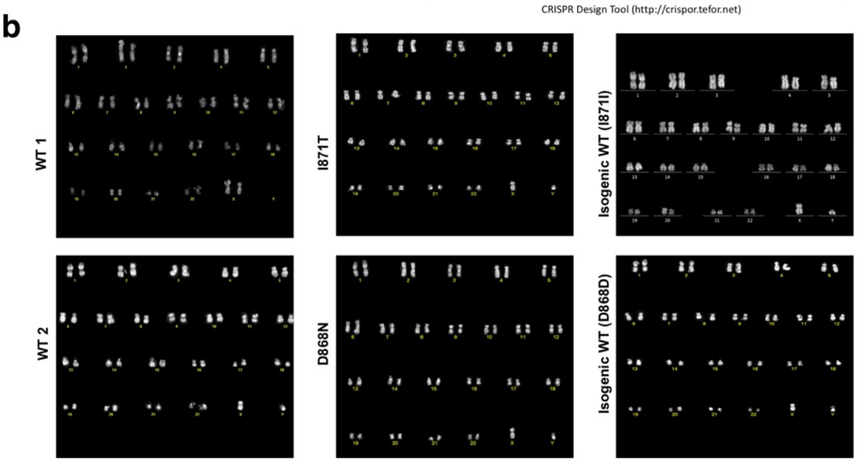

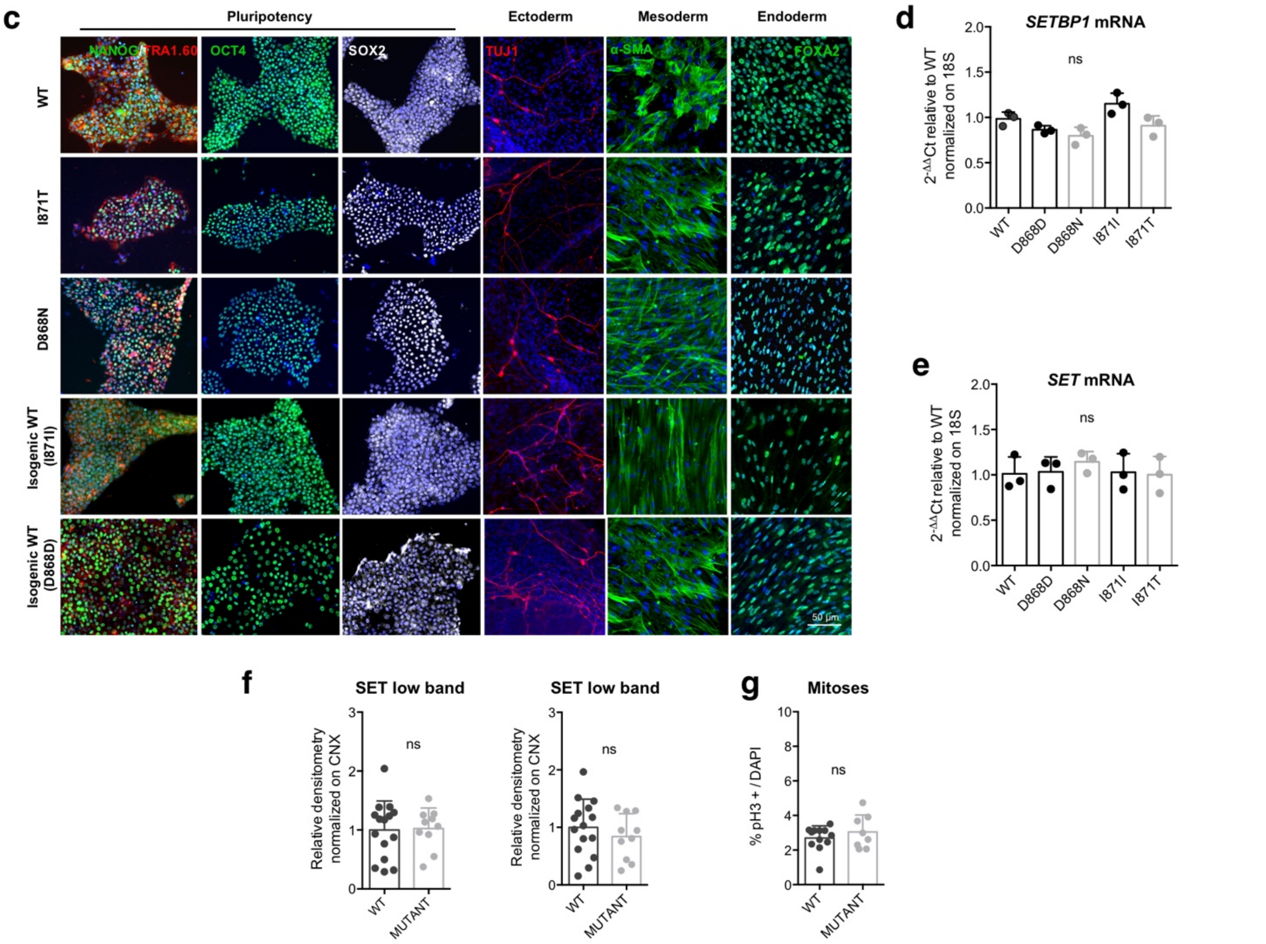

Supplementary Fig. 1 SGS iPSCs do not display phenotypic abnormalities. a ssDNA WT donor and CRISPR/Cas9 sgRNAs on SETBP1 mutational hotspot (left). Five most likely potential off target genomic sites falling in coding regions for sgRNAs selected for mutation correction (sgI871T #9 and sgD868N #6 for pt 1 and pt 2, respectively), in all cases with four nucleotide mismatches between the sgRNA and the eventual off-target sequence (highlighted in red, PAM domains in green) (right). **b** Representative images of karyotype analysis of WT, mutated and isogenic corrected control iPSCs. **c** Representative immunofluorescence images of iPSC clones expressing the pluripotency markers NANOG, TRA-1-60, OCT4 and SOX2 (left). Immunofluorescence analysis of multi-germ layer differentiation of iPSCs. Samples are positive and homogeneous for differentiation markers of the three different germ layers, TUJ1 (βIII-TUBULIN) for ectoderm, α-SMA for mesoderm and FOXA2 for endoderm (right). Images taken at the same magnification. **d** Quantitative RT-PCR analysis of *SETBP1* mRNA in WT, isogenic control (D868D and I871I) and mutant (D868N and I871T) iPSCs: WT vs. D868D p=0.5166; WT vs. D868N p=0.1610; WT vs. I871I p = 0.2457; WT vs. I871T p = 0.8395; D868D vs. D868N p= 0.8905, I871I vs. I871T p = 0.0534. One-way ANOVA followed by Tukey post-hoc test for multiple comparisons. n=3. **e** Quantitative RT-PCR analysis of *SET* mRNA inWT, isogenic control and mutant iPSCs. WT vs. D868D p=0.9998; WT vs. D868N p=0.8823; WT vs. I871I p>0.9999; WT vs. I871T p>0.9999; D868D vs. D868N p=0.9350, I871I vs. I871T p =0.9997. One-way ANOVA followed by Tukey post-hoc test for multiple comparisons. n=3. **f** Merged quantification of higher and lower bands of SET immunoblotting (see Fig. 1d), WT vs. MUT High band: p=0.8860; Low band: p=0.3996, two-sided unpaired t test. n=5. **g** Merged quantification of mitotic figures as from pH3 staining (see Fig. 1g), WT vs. MUT p = 0.3649, two-sided unpaired t test. n=4. All data expressed as mean ± SEM. ns: non-significant differences when p>0.05.

Supplementary Figure 2

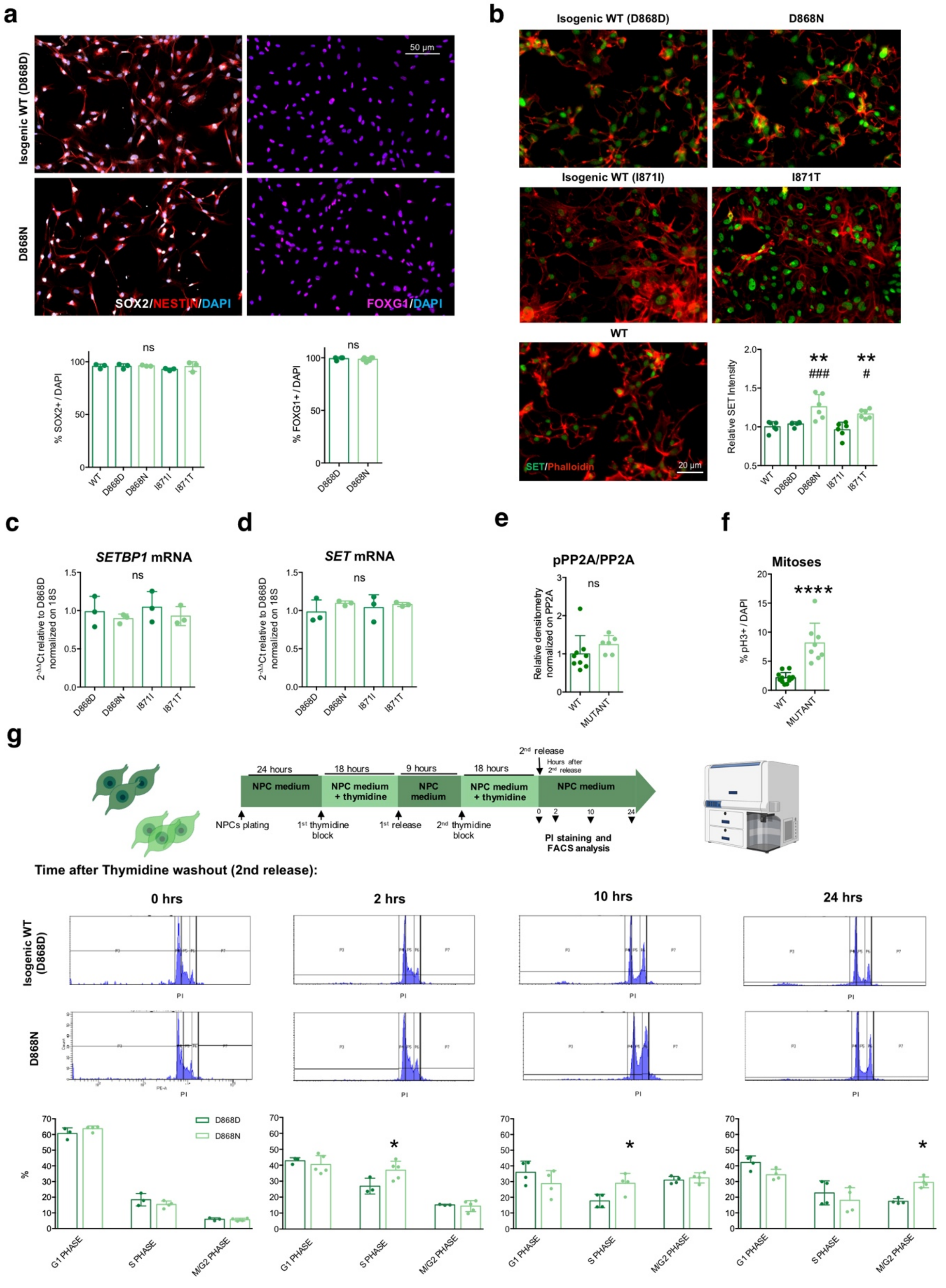

Supplementary Fig. 2 SGS NPC additional characterization. a Representative images (taken at the same magnification) of immunostaining for the neural progenitor markers, SOX2 (p=0.5367, one-way ANOVA followed by Tukey post-hoc test for multiple comparisons, n=3) and telencephalon

marker FOXG1 (p=0.5532, two-sided unpaired t test, n=4) and relative quantifications. **b** Immunofluorescence analysis and quantification of SET relative intensity in NPCs. * indicates statistic test between isogenic cell line pairs, # refers to comparison between each mutant cell line with the WT. D868D vs. D868N **p=0.0030, I871I vs. I871T **p=0.0070; WT vs D868N ###p=0.0006; WT vs I871T #p=0.0373. One-way ANOVA followed by Tukey post-hoc test for multiple comparisons. n=6. Images taken at the same magnification. **c** Quantitative RT-PCR analysis of *SETBP1* mRNA in isogenic control (D868D and I871I) and mutant (D868N and I871T) NPCs. p=0.6755. One-way ANOVA followed by Tukey post-hoc test for multiple comparisons. n=3. **d** Quantitative RT-PCR analysis of *SET* mRNA in isogenic control and mutant NPCs. p=0.6520. One-way ANOVA followed by Tukey post-hoc test for multiple comparisons. n=3. **e** Merged quantification of pPP2A/PP2A ratio (see Fig. 2e), WT vs. MUT p=0.3593, two-sided unpaired t test. n=5. **f** Merged quantification of mitotic figures as from pH3 staining (see Fig. 2h), WT vs. MUT ****p <0.0001, two-sided unpaired t test. n=4. **g** Cell cycle analysis by PI staining and FACS quantification after synchronization with double thymidine block (upper part); percentage of cells present in each phase in D868D and D868N cells (n=3) in the indicated time point after thymidine release. Two-way ANOVA followed by Sidak's post-hoc test for multiple comparisons. See Supplementary Dataset 3 for statistical details. All data expressed as mean ± SEM. ns: non-significant differences when p>0.05; *p<0.05, **p<0.01, ***p<0.001, ****p<0.0001.

Supplementary Figure 3

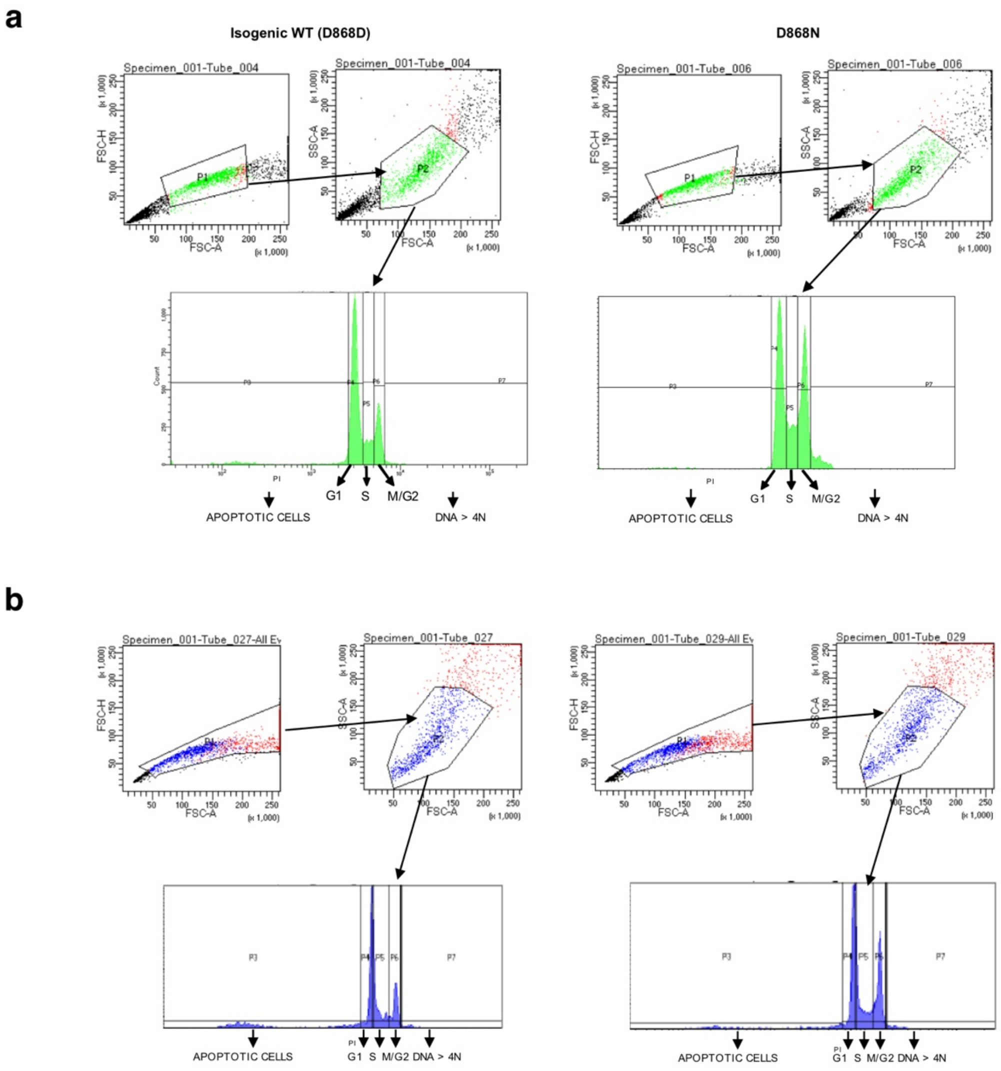

Supplementary Fig. 3 Gating strategies used for FACS analysis. FSC-A/FSC-H were used to exclude doublets and FSC-A/SSC-A were used to exclude debris. Cell count were identified on P2 gate by propidium iodide staining and gates that identify different cell cycle phases were set according to peak distribution (P3: apoptotic cells; P4: G1-phase; P5: S-phase; P6: M/G2-phase; P7: cells with DNA content > 4N). **a** Gating panels relative to Fig 3c and Supplementary Fig. 6b. **b** Gating panels relative to Supplementary Fig. 2g.

Supplementary Figure 4

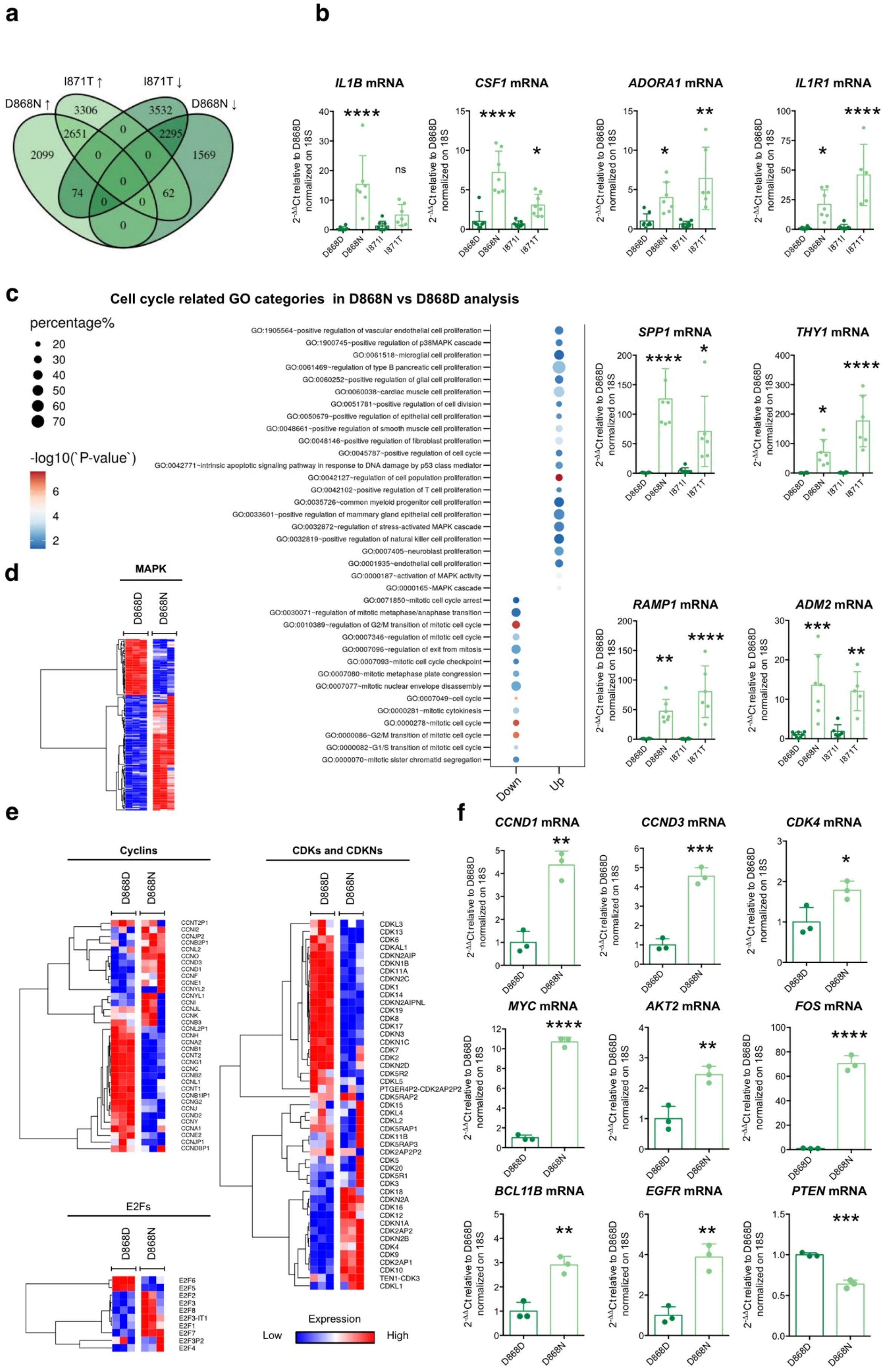

Supplementary Fig. 4 Transcriptional dysregulation upon SGS mutation. a Venn diagram showing the overlap between those genes being differentially expressed in D868N, or I871T vs. D868D control NPCs. **b** Independent validation of deregulated genes belonging to non-neural lineages through RT-qPCR analysis. *IL1B:* D868D vs. D868N ****p<0.0001, I871I vs. I871T p=0.5995; *CSF1:* D868D vs. D868N ****p <0.0001, I871I vs. I871T *p=0.0412; *ADORA1:* D868D vs. D868N *p = 0.0471, I871I vs. I871T ***p=0.0001; *IL1R1:* D868D vs. D868N *p = 0.02131, I871I vs. I871T ****p<0.0001; *SPP1:* D868D vs. D868N ****p<0.0001, I871I vs. I871T *p=0.0130; *THY:* D868D vs. D868N *p=0.0277, I871I vs. I871T ****p<0.0001; *RAMP1:* D868D vs. D868N **p=0.0021, I871I vs. I871T ****p<0.0001; *ADM2:* D868D vs. D868N ***p=0.0002, I871I vs. I871T **p=0.0081. One-way ANOVA followed by Tukey post-hoc test for multiple comparisons. n≥6 differentitiation experiments. **c** Heatmap showing functional enrichment statistical significance of gene ontology categories related to cell cycle regulation, progenitor cell proliferation, and MAPK pathway. **d** Gene expression heat-map of deregulated MAPK-related genes in D868N vs. D868D NPCs. **e** Gene expression heat-maps deregulated genes within families of cyclins, CDKs/CDKNs and E2Fs factors in D868N vs. D868D NPCs. **f** Independent validation of deregulated genes through RT-qPCR analysis. *CCND1* p=0.0017; *CCND3* p=0.0003; *CDK4* p=0.0331; *MYC* p <0.0001, *AKT2* p= 0.0067, *FOS* p <0.0001, *BCL11A* p= 0.0028, *EGFR* p= 0.0030, *PTEN* p= 0.0003. Two-sided unpaired t test. n=3. Data expressed as mean ± SEM. *p<0.05, **p<0.01, ***p<0.001, ****p<0.0001.

Supplementary Figure 5

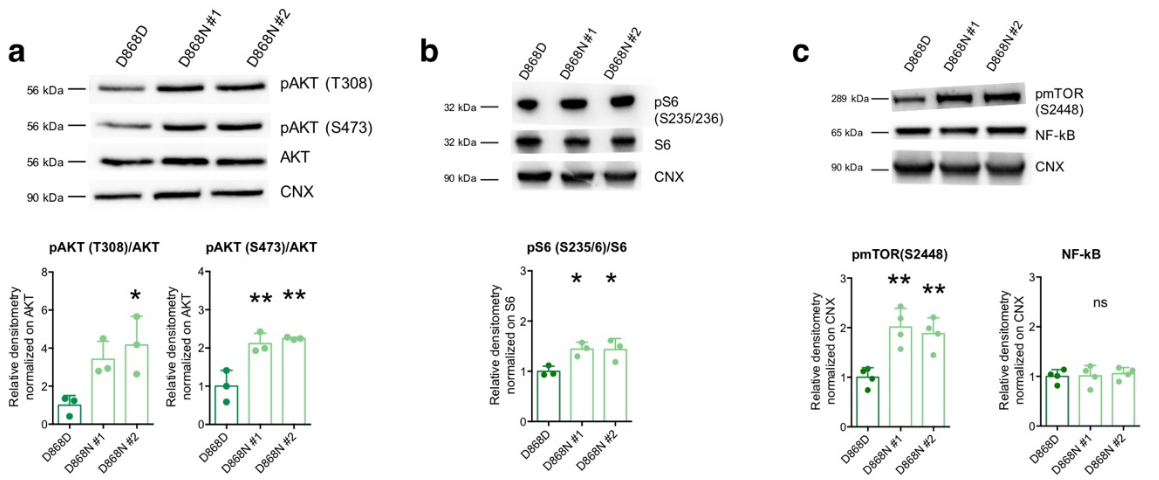

Supplementary Fig. 5 AKT signaling in SGS NPCs. **a** Immunoblotting for total AKT and phosphorylated AKT (pAKT Tyr308 p=0.0261 and pAKT Ser473 p= 0.0032) in isogenic control and mutant NPCs and relative quantification as pAKT/AKT ratio. One-way ANOVA followed by Tukey post-hoc test for multiple comparisons. n=3. **b** Immunoblotting for total S6 and phosphorylated S6 (pS6 Ser 235/S236) and relative quantification of two independent clones, #1 and #2: D868D vs. D868N #1 *p=0. 0353, D868D vs. D868N #2 *p=0.0387, one-way ANOVA followed by Tukey's post-hoc test for multiple comparisons. n=3. **c** Immunoblotting for total phosphorylated mTOR (pmTOR Ser 2448) and NF-kB and relative quantification of two independent clones, #1 and #2: pmTOR: D868D vs. D868N #1 **p=0. 0030, D868D vs. D868N #2 **p=0.0072; NF-kB: D868D vs. D868N #1 p=0.9954, D868D vs. D868N #2 p=0.9122; one-way ANOVA followed by Tukey's post-hoc test for multiple comparisons. n=4. Data expressed as mean ± SEM. *p<0.05, **p<0.01.

Supplementary Figure 6

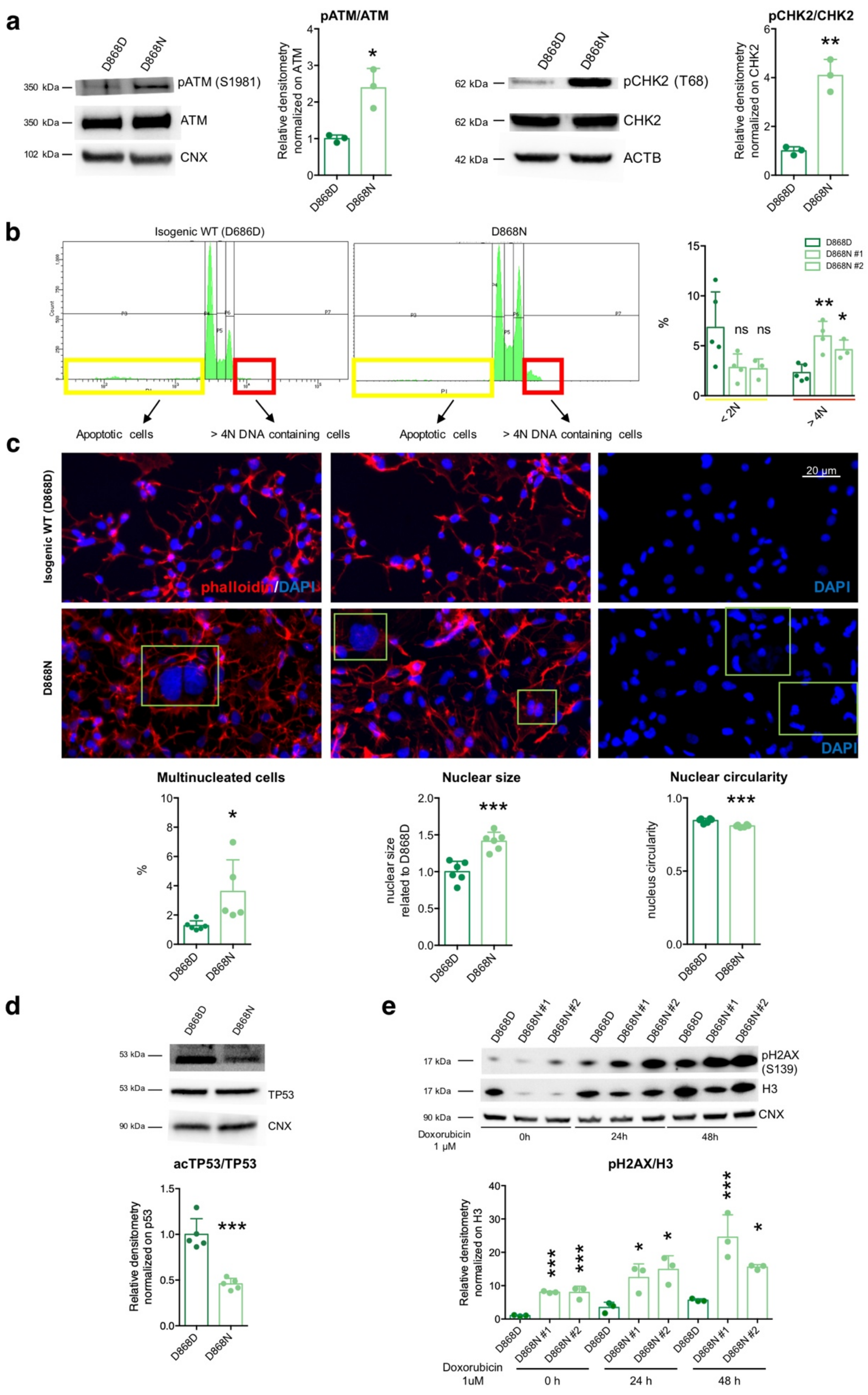

Supplementary Fig. 6 Cancer-like features of SGS NPCs. a Immunoblotting for phosphorylated and total forms of both ATM (pATM, Ser1981) and CHK2 (pCHK2, Thr68) and relative quantifications (pATM/ATM p=0.0113; pCHK2/CHK2 p=0.0014) in isogenic control (D868D) and mutant (D868N) NPCs. two-sided unpaired t test. n=3. **b** PI staining and FACS analysis of NPCs showing decreased cell death (highlighted in yellow, p=0.0655) and increased polyploidy in mutant (D868N) cells (highlighted in red, p=0.0026). n=3 independent experiments. One-way ANOVA followed by Tukey post-hoc test for multiple comparisons. **c** Representative immunofluorescence images (taken at the same magnification) and quantification showing anomalous nuclear size (p=0.0003) and shape (p=0.0005) and presence of multinucleated cells (p=0.0270) in mutant (D868N) cultures compare to control (D868D). One-way ANOVA followed by Tukey post-hoc test for multiple comparisons. n=6. **d** Immunoblotting for total and acetylated TP53 (acTP53 Lys382) and relative quantification of acTP53/TP53 ratio in isogenic control (D868D) and mutant (D868N) NPCs. (p=0.0002). Two-sided unpaired t test. n=5. **e** DNA damage accumulation due to 1 μM doxorubicin treatment over time (indicated) detected by immunoblotting of pH2AX in isogenic control (D868D) and mutant (D868N) NPCs. Statistical comparison between control and mutants within time points are shown. One-way ANOVA followed by Tukey post-hoc test for multiple comparisons. n=3. See Supplementary Dataset 3 for details of statistical analysis. All data expressed as mean ± SEM. ns: non-significant differences when $p>0.05$; *$p<0.05$, **$p<0.01$, ***$p<0.001$, ****$p<0.0001$.

Supplementary Figure 7

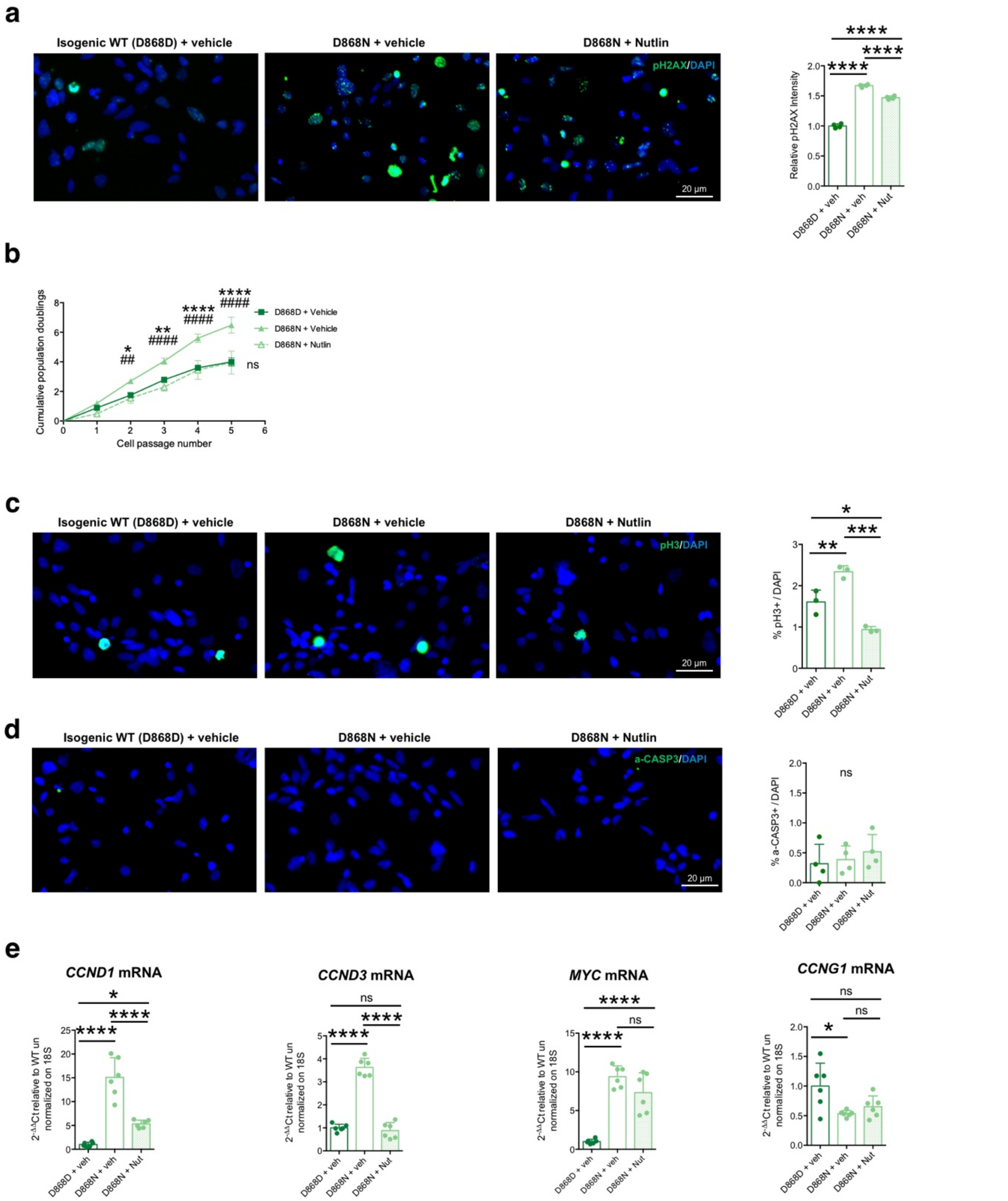

Supplementary Fig. 7 Nutlin treatment in SGS NPCs. a Phosphorylated H2AX (pH2AX Ser139) immunostaining and quantification in isogenic control (+vehicle) and mutant NPCs (+vehicle or Nutlin-3a). D868D+veh vs. D868N+veh ****$p<0.0001$, D868D+veh vs. D868N+Nut ****$p<0.0001$, D868N+veh vs. D868N+Nut ****$p<0.0001$. One-way ANOVA followed by Tukey's post-hoc test for multiple comparisons. n=4. Images taken at the same magnification. **b** Growth curve analysis of isogenic control (+Vehicle) and mutant NPCs (+Vehicle and +Nutilin-3a). Two-way ANOVA followed by Tukey post-hoc test for multiple comparisons. n=3. See Supplementary Dataset

3 for statistical analysis. **c** Phosphorylated H3 (pH3 Ser10) immunostaining and quantification in isogenic control (+vehicle) and mutant NPCs (+vehicle or Nutlin-3a) and relative quantification. D868D+veh vs. D868N+veh **p=0.0078, D868D+veh vs. D868N+Nut *p =0.0122, D868N+veh vs. D868N+Nut ***p=0.0003. One-way ANOVA followed by Tukey's post-hoc test for multiple comparisons. n=3. Images taken at the same magnification. **d** Activated-CASPASE 3 (cleaved at Asp175) immunostaining and relative quantification in isogenic control (+vehicle) and mutant NPCs (+vehicle or Nutlin-3a). p=0.6187. One-way ANOVA followed by Tukey's post-hoc test for multiple comparisons. n=4. Images taken at the same magnification. **e** RT-qPCR analysis of deregulated genes associated with cell cycle/proliferation in isogenic control (+vehicle) and mutant NPCs (+vehicle or Nutlin-3a) through. *CCND1:* D868D+veh vs. D868N+veh ****p<0.0001, D868D+veh vs. D868N+Nut *p= 0.0212, D868N+veh vs. D868N+Nut ****p<0.0001. *CCND3:* D868D+veh vs. D868N+veh **** p<0.0001, D868D+veh vs. D868N+Nut p=0.8018, D868N+veh vs. D868N+Nut ****p<0.0001. *MYC:* D868D+veh vs. D868N+veh ****p<0.0001, D868D+veh vs. D868N+Nut ****p<0.0001, D868N+veh vs. D868N+Nut p=0.1202. *CCNG1:* D868D+veh vs. D868N+veh *p=0.0139, D868D+veh vs. D868N+Nut p=0.0674, D868N+veh vs. D868N+Nut p=0.6980. One-way ANOVA followed by Tukey's post-hoc test for multiple comparisons. n=3.All data expressed as mean ± SEM. ns: non-significant differences when p>0.05; *p<0.05, **p<0.01, ***p<0.001, ****p<0.0001.

Supplementary Figure 8

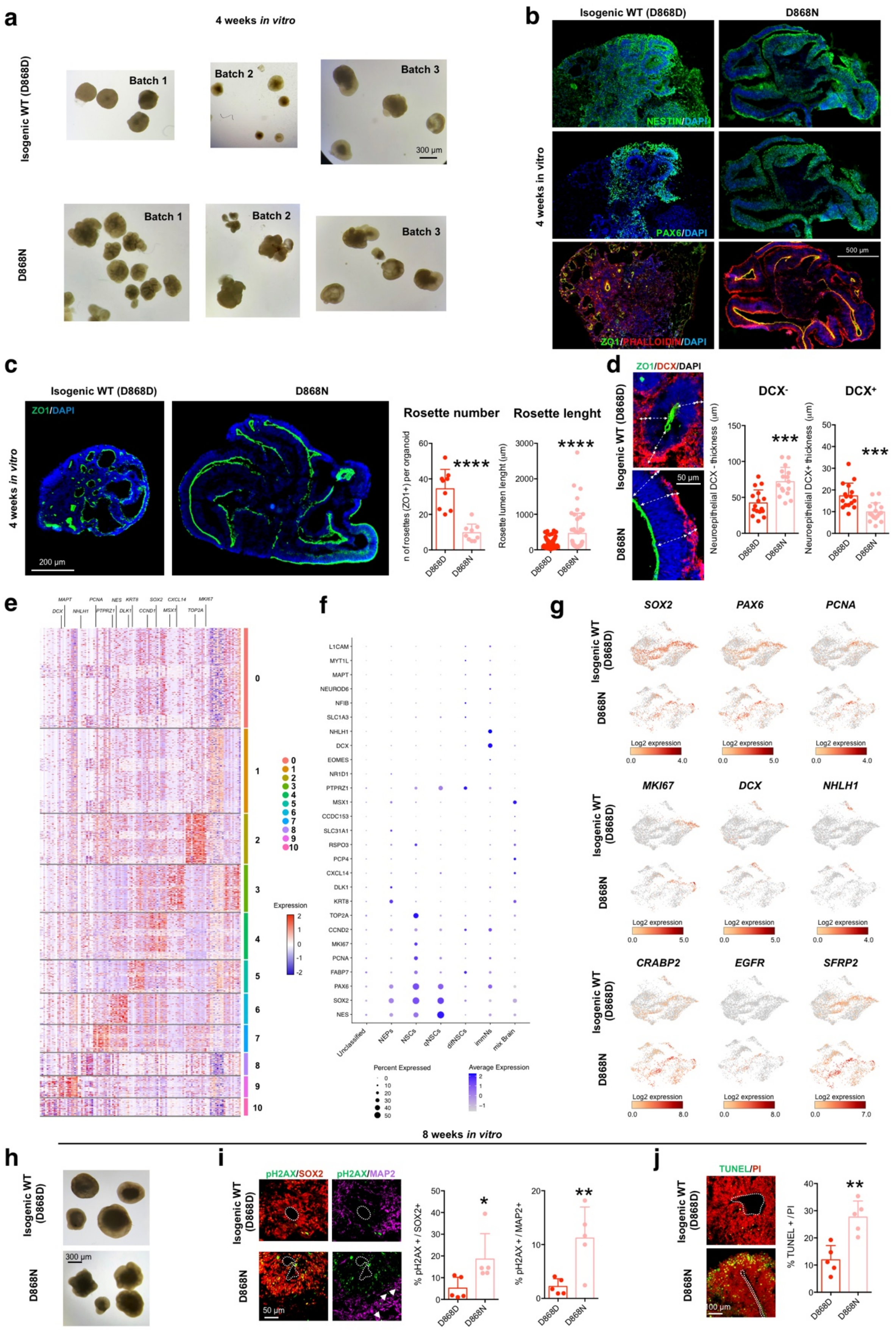

Supplementary Fig. 8 SGS phenotype in cortical organoids. a Representative bright-field images of both control and mutant 4-week-old organoids from 3 different batches. **b** Representative immunofluorescence images (Images taken at the same magnification) of cortical progenitor markers NESTIN and PAX6 structural tight-junction maker ZO1, and phalloidin staining of cryo-sections of isogenic control and mutant cerebral organoids. **c** Quantification of $ZO1^+$ neuroepithelial-like structures (rosettes) in terms of number per organoid (****$p< 0.0001$, two-sided unpaired t test) and length of the lumen per rosette (****$p< 0.0001$, two-sided unpaired t test). Rosette number: b=3 (batches), o=9 (organoids). Rosette lumen length: b=3 (batches), o=3 (organoids), v≥47 (v=ventricles). Images taken at the same magnification. **d** Evaluation of the neuroepithelium thickness (as DCX^- and DCX^+ territories) of the rosettes (DCX^- ***$p=0.0001$; DCX^+ ***$p=0.0003$; two-sided unpaired t test. b=3 (batches), o=6 (organoids), v=16 (v=ventricles). Images taken at the same magnification. **e** Representative heatmap of genes associated with identified clusters (see also Supplementary Dataset 1). **f** Dot plot showing category-specific gene expression across categories. **g** UMAP plots of both control and SGS organoids coloured by expression level of selected marker genes (log2 expression). **h** Representative bright-field images (taken at the same magnification) of both control and mutant 8-week-old organoids. **i** Immunostaining for phosphorylated histone H2AX (pH2AX Ser139, marker of DNA DSBs), SOX2 and MAP2 in rosettes of both isogenic control and mutant 8-weeks-old organoids. Quantification: pH2AX/SOX2 *$p=0.0471$, pH2AX/MAP2 **$p=0.0098$, two-sided unpaired t test. Dashed line indicates ventricular surface. Arrowheads indicate co-localization between pH2AX and MAP2. b=3 (batches), o=3 (organoids), v=5 (ventricles). Images taken at the same magnification. **j** TUNEL assay counterstained with propidium iodide (PI) in rosettes of both isogenic control and mutant 8-weeks-old organoids (**$p<0.0022$, two-sided unpaired t test). Dashed line indicates ventricular surface. b=3 (batches), o=3 (organoids), v=5 (ventricles). Images taken at the same magnification. All data expressed as mean ± SEM. ns: non-significant differences when $p>0.05$; *$p<0.05$, **$p<0.01$, ***$p<0.001$, **** $p<0.0001$.

Supplementary Figure 9

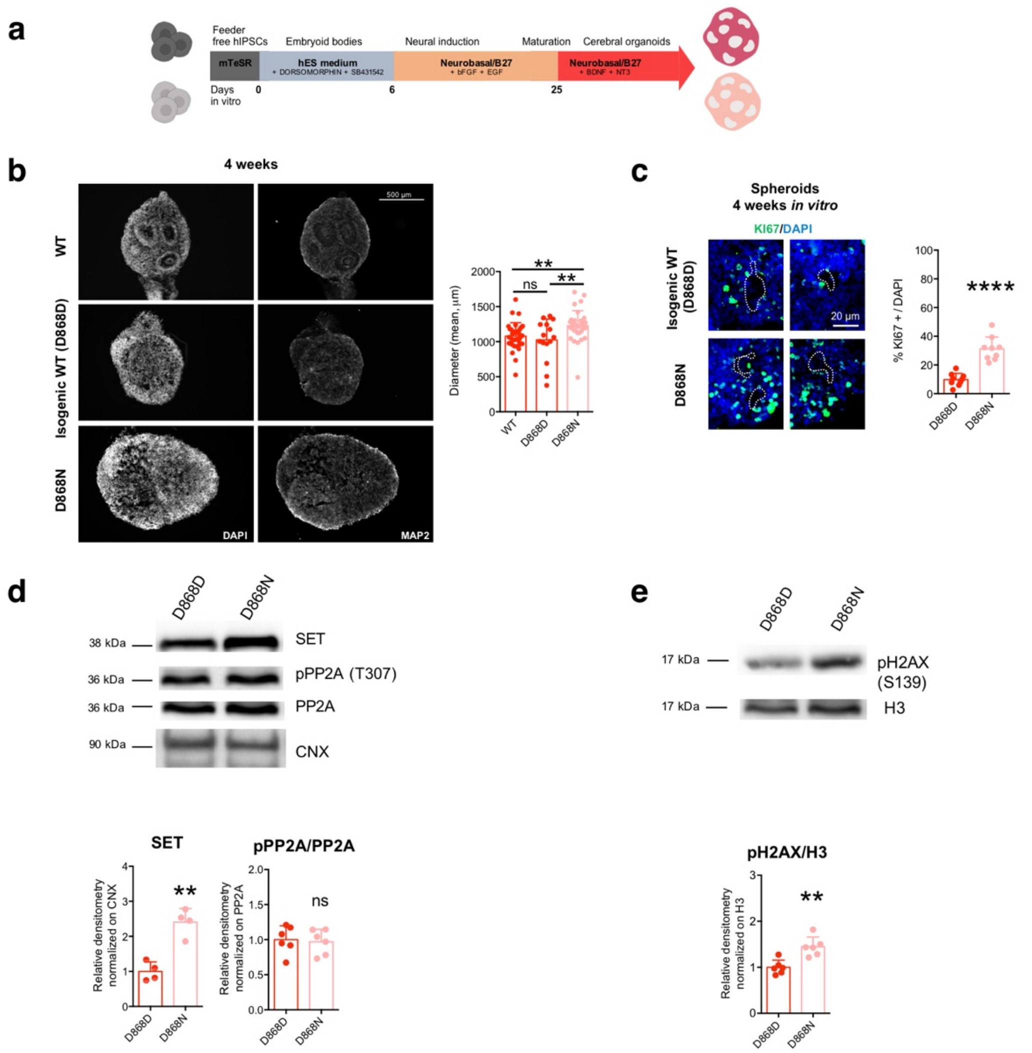

Supplementary Fig. 9 SGS phenotype in cortical spheroids. a Scheme for cortical spheroid generation protocol. **b** Representative immunofluorescence images (taken at the same magnification) of cortical spheroids from both unrelated WT, isogenic control (D868D) and mutant (D868N) cells, stained for MAP2 neuronal marker (left) and quantification of spheroid size (right). WT vs. D868D p=0.7073; WT vs. D868N **p=0.0187; D868D vs. D868N **p=0.0116. One-way ANOVA followed by Tukey post-hoc test for multiple comparisons. b=3 (batches), o≥15 (organoids). **c** Immunostaining for KI67 counterstained with DAPI in rosettes of both isogenic control and mutant 4-weeks-old spheroids (****p<0.0001, two-sided unpaired t test). Dashed line indicates ventricular surface. b=1 (batches), o=3 (organoids), v=9 (ventricles). Images taken at the same magnification. **d** SET-PP2A axis in cortical spheroids; immunoblotting analysis revealed SET (**p=0.0010, two-sided unpaired t test, n=4) protein accumulation in mutant spheroids, which did not correlate with PP2A activity inhibition (pPP2A/PP2A ratio p= 0.7798, two-sided unpaired t test, n=6). **e** Immunoblotting for

pH2AX (Ser139) and relative quantification showing increased presence of DNA damage in SGS mutant spheroids. (**p=0.0019, two-sided unpaired t test). n=6.

All data expressed as mean ± SEM. ns: non-significant differences when p>0.05; *p<0.05, **p<0.01, ***p<0.001, **** p<0.0001. Images, immunostaining and immunoblotting were analyzed at 4 weeks of differentiation cortical spheroids.

Supplementary Figure 10

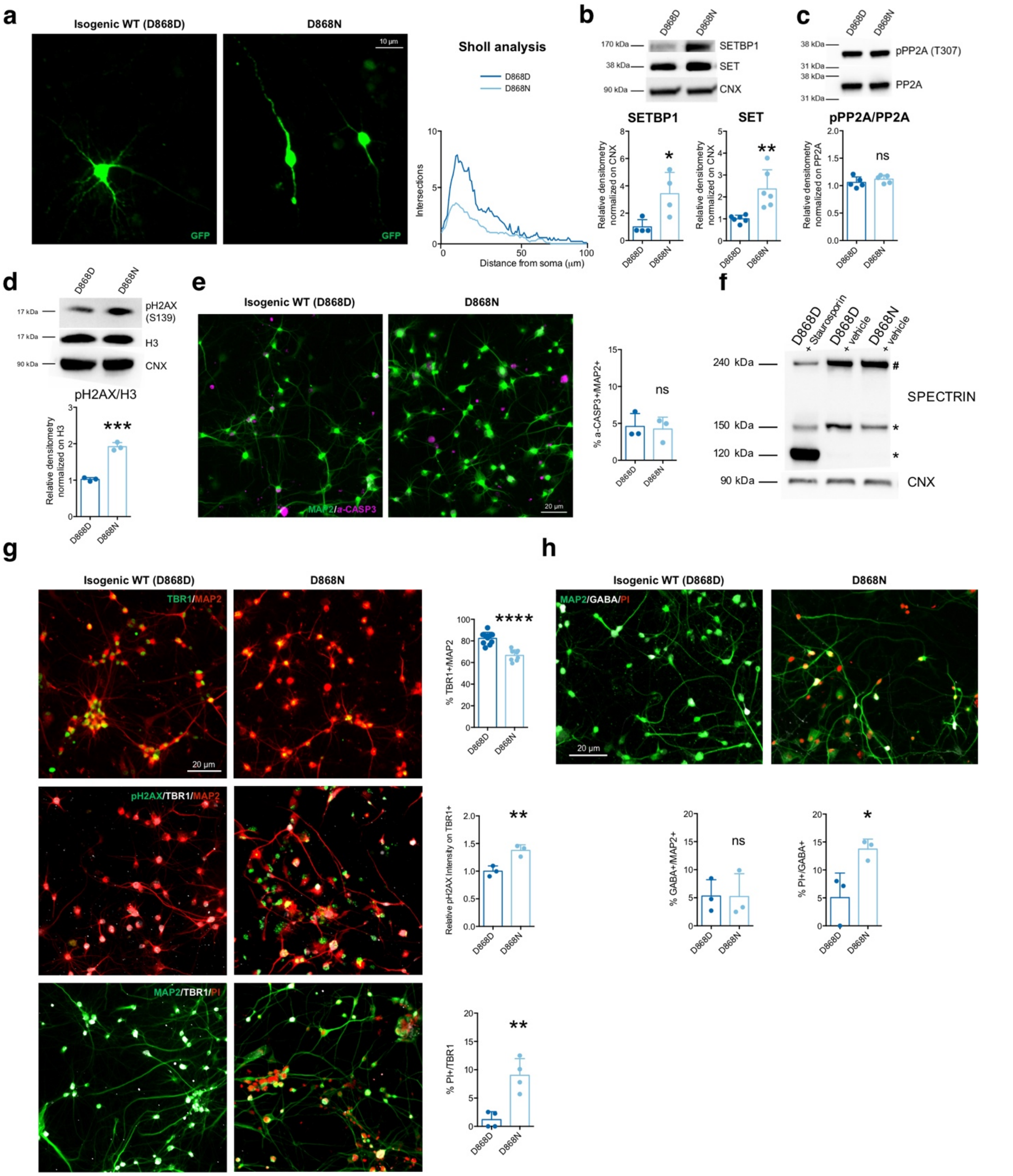

Supplementary Fig. 10 Additional characterization of SGS neurons. a Representative images (taken at the same magnification) of isogenic control (D868D) and mutant (D868N) NPC-derived neurons (left), and Sholl analysis (right). $p<0.0001$. Two-way ANOVA. n=10 cells from 3 independent experiments. **b** SETBP1 (n=4) and SET (n=6) immunoblotting in isogenic control and mutant NPC-derived neurons and relative quantification. SETBP1 *$p=0.0255$; SET **$p=0.0038$.

Two-sided unpaired t test. **c** Total and phosphorylated PP2A (pPP2A Tyr307) immunoblotting in isogenic control and mutant NPC-derived neurons and relative quantification of pPP2A/PP2A ratio. p=0.3075 two-sided unpaired t test. n=5. **d** pH2AX (Ser139) immunoblotting and relative quantification in isogenic control and mutant NPC-derived neurons. ***p=0.0002. Two-sided unpaired t test. n=3. **e** Representative images (taken at the same magnification) of MAP2 and active-CASPASE3 immunostaining and relative quantification of a-CASPASE^{+}/MAP2^{+} cells in isogenic control and mutant NPC-derived neuronal cultures. p=0.8109. Two-sided unpaired t test. n=3. **f** SPECTRIN immunoblotting in isogenic control and mutant NPC-derived neuronal cultures. #: full length *: cleavage product. D868D progenitors treated with 2μM Staurosporin for 12 hrs were added as positive control for the assay. **g** Representative images (taken at the same magnification) of TBR1, MAP2, phosphorylated histone H2AX (pH2AX Ser139), and PI immunostaining and relative quantification of both TBR1^{+}/MAP2^{+} (n=8), pH2AX^{+}/TBR1^{+} (n=3) and PI^{+}/TBR1^{+} (n=4) in isogenic control and mutant NPC-derived neuronal cultures. TBR1/MAP2: ****p<0.0001; pH2AX/TBR1: **p=0.0094; PI/TBR1: **p=0.0029. Two-sided unpaired t test. **h** Representative images (taken at the same magnification) of MAP2, GABA, and PI immunostaining and relative quantification of both GABA^{+}/MAP2^{+}, and PI^{+}/GABA^{+}, in isogenic control and mutant NPC-derived neuronal cultures. GABA/MAP2: p=0.9777; PI/GABA: *p=0.0340. Two-sided unpaired t test. n=3.All data expressed as mean ± SEM. ns: non-significant differences when p>0.05; *p<0.05, **p<0.01, ***p<0.001, **** p<0.0001. Analysis were performed at 4 weeks of neuronal differentiation.

Supplementary Figure 11

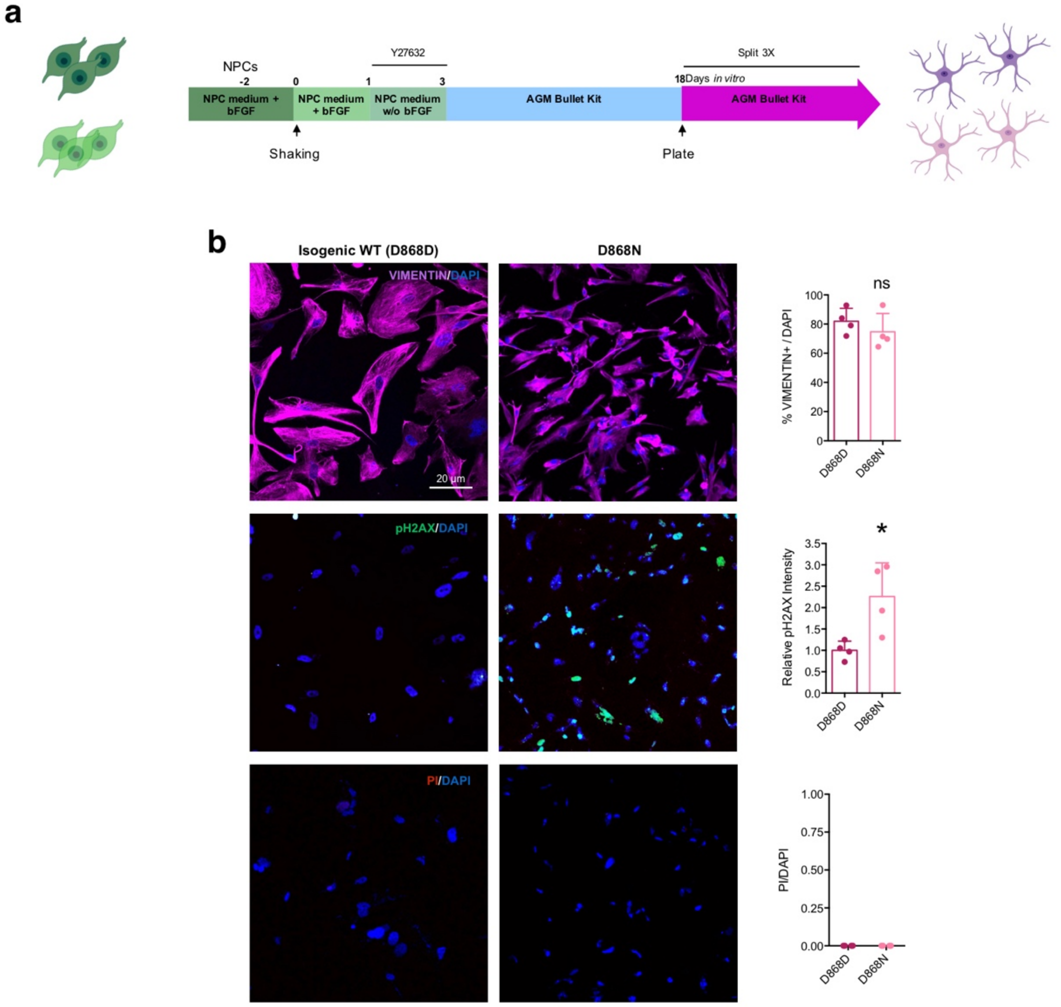

Supplementary Fig. 11 SGS astrocytes characterization. a NPC-derived astrocytic differentiation protocol. **b** Representative images (taken at the same magnification) of VIMENTIN (up), phosphorylated histone H2AX (pH2AX Ser139, marker of DNA DSBs) (middle), and PI (bottom) immunostaining, counterstained with DAPI, and relative quantification in isogenic control (D868D) and mutant (D868N) NPC-derived astrocytic cultures. VIMENTIN/DAPI: p=0.3833; pH2AX/DAPI: p=0.0216. Two-sided unpaired t test. PI/DAPI: no statistical analysis since no PI^+ cells were found. n=4 coverslips. All data expressed as mean ± SEM. ns: non-significant differences when p>0.05; *p<0.05.

Supplementary Figure 12

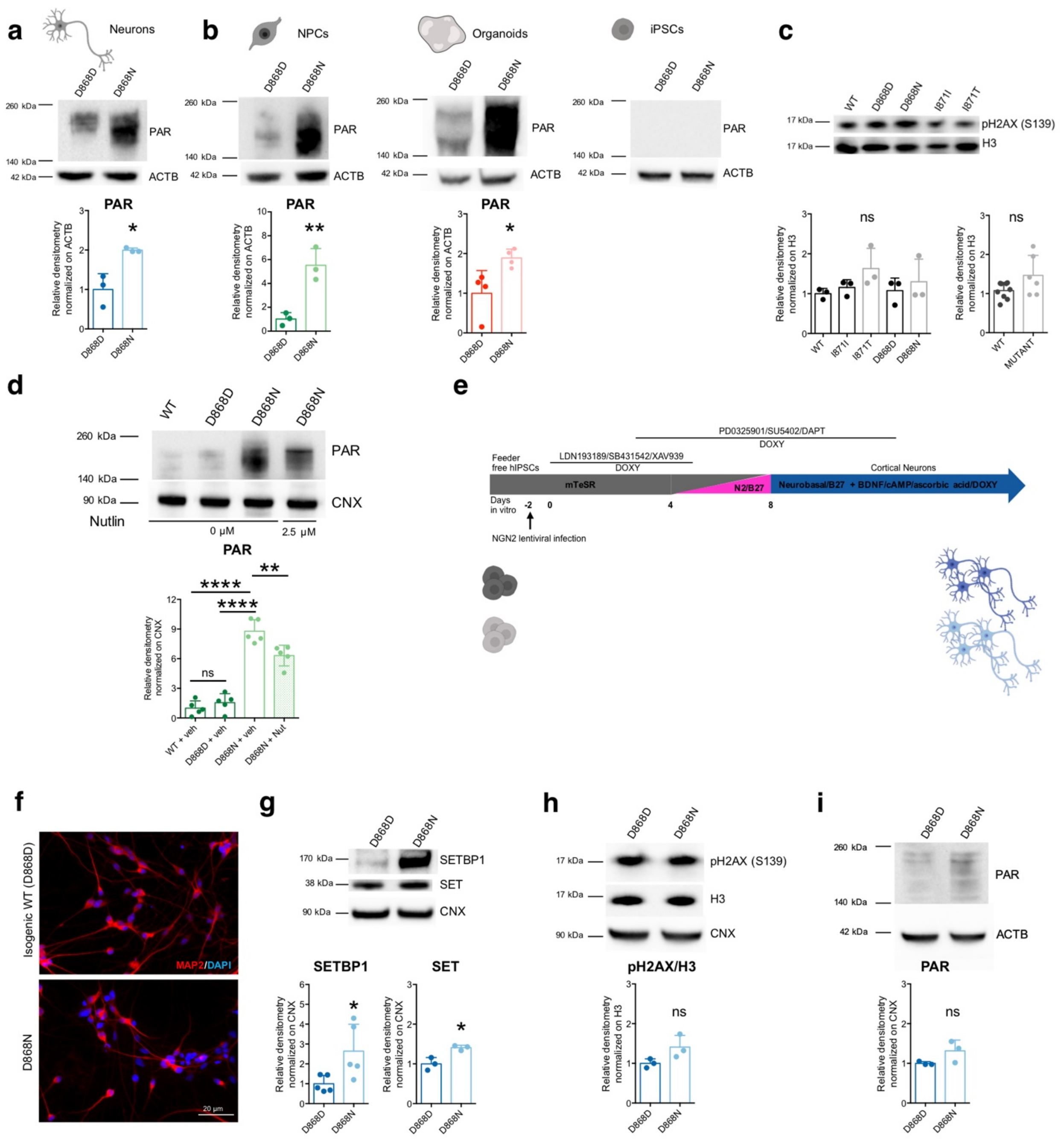

Supplementary Fig. 12 PAR levels in iPSC and neural derivatives. a and b Immunoblotting for PAR polymers and relative quantification in isogenic control and mutant NPC-derived neurons (**a,** p=0.0130, n=3)**,** NPCs (**b,** left, **p=0.0064, n=3), cerebral organoids (**b,** middle**,** *p=0.0277, n=4) and iPSCs (**b,** right, n=3). For NPC-derived neurons, NPCs and cerebral organoids, significance was calculated by two-sided unpaired t test. For iPSCs, no statistical analysis was performed, since their parylation levels were undetectable. **c** Immunoblotting for pH2AX (Ser139) and relative quantification in WT, isogenic control (D868D and I871I) and mutant (D868N and I871T) iPSCs. p=0.3471. One-way ANOVA followed by Tukey post-hoc test for multiple comparisons. WT vs.

MUT: p=0.0606. Two-sided unpaired t test. n=3. **d** Immunoblotting for PAR polymers and relative quantification in WT, D868D, D868N and D868N + Nutlin-3a NPCs and relative quantification. WT+veh vs. D868D+veh p=0.8073; WT+veh vs. D868N+veh ****p <0.0001; D868D+veh vs. D868N+veh ****p<0.0001; D868N+veh vs. D868N+veh **p=0.0053. One-way ANOVA followed by Tukey's post-hoc test for multiple comparisons. n=5. **e** Differentiation protocol of iPSC-derived neurons. **f** Representative images (taken at the same magnification) of MAP2 immunostaining in isogenic control (D868D) and mutant (D868N) iPSC-derived neuronal cultures. **g** Immunoblotting for SETBP1 (n=5) and SET (n=3) in isogenic control and mutant iPSC-derived neurons and relative quantification. SETBP1 *p=0.0316; SET *p=0.0144. Two-sided unpaired t test. **h** Immunoblotting for pH2AX (Ser139) and relative quantification in isogenic control and mutant iPSC-derived neurons. p=0.0881. Two-sided unpaired t test. n=3. **i** Immunoblotting for PAR polymers and relative quantification in isogenic control and mutant iPSC-derived neurons. p=0.1201. Two-sided unpaired t test. n=3. All data are expressed as mean ± SEM. ns: non-significant differences when p>0.05; *p<0.05, **p<0.01, ****p<0.0001. Analysis were performed at 4 weeks of neuronal differentiation.

Supplementary Figure 13

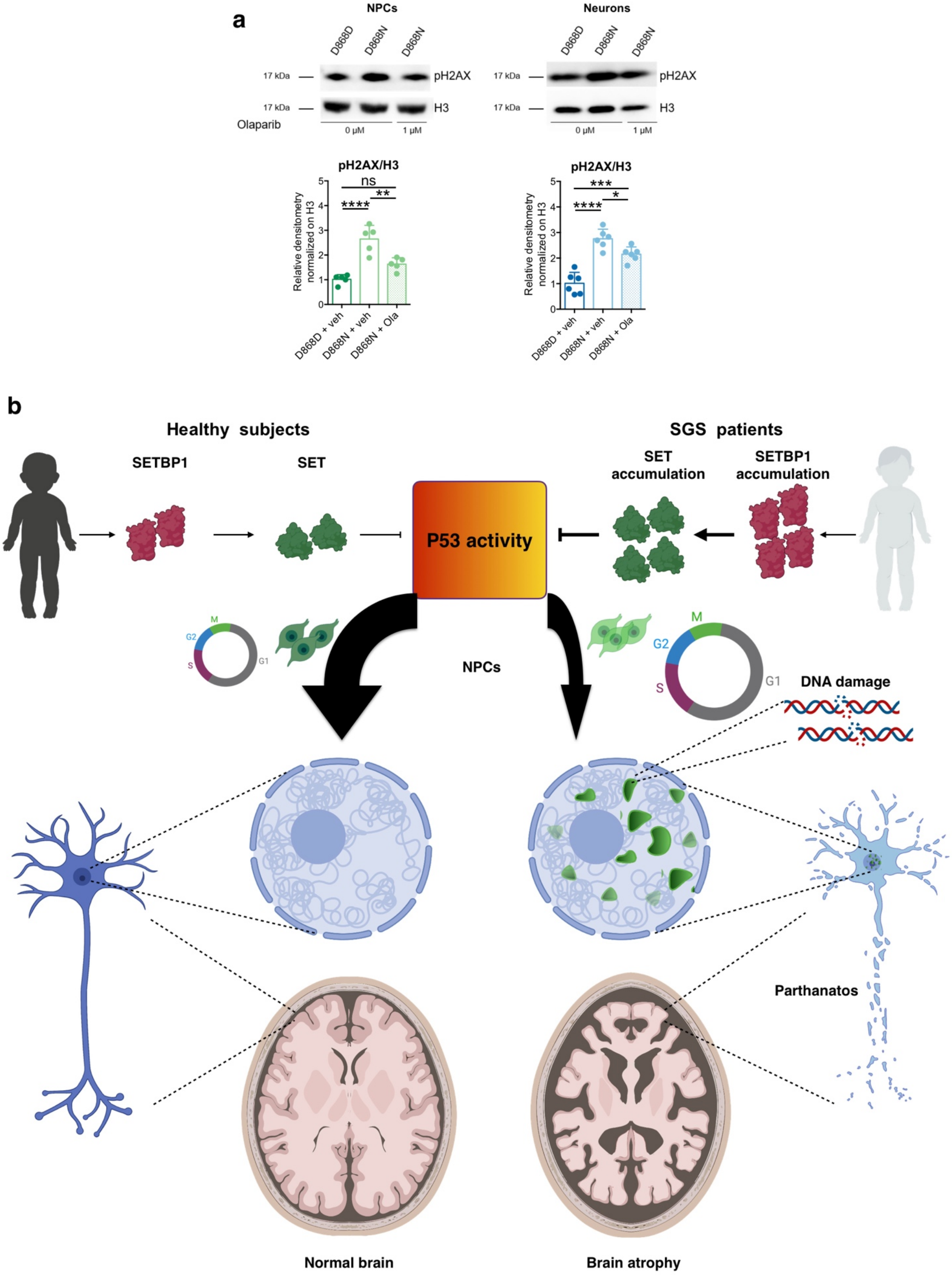

Supplementary Fig. 13 Olaparib rescue in NPCs and Neurons and schematic model. a Immunoblotting for pH2AX and relative quantification in mock-treated isogenic control (D868D), mock-treated mutant (D868N) and Olaparib-treated mutant (D868N)lines, either NPCs (left; D868D+Vehicle vs. D868N+Vehicle ****p<0.0001; D868D+Vehicle vs. D868N+Olaparib p=0.0534; D868N+Vehicle vs. D868N+Olaparib **p=0.0026, n=5) or NPC-derived neurons (4 weeks of differentiation) (right; D868D+Vehicle vs. D868N+Vehicle ****p <0.0001; D868D+Vehicle vs. D868N+Olaparib ***p=0.0002; D868N+Vehicle vs. D868N + Olaparib *p=0.0318, n=6.). Differences were assessed using one-way ANOVA followed by Tukey post-hoc test for multiple comparisons. All data expressed as mean ± SEM. ns: non-significant differences when p > 0.05; *p < 0.05, **p < 0.01, ***p < 0.001, ****p < 0.0001. **b** Schematic representation of a model of pathological mechanism in SGS generating from our data.

Supplementary Figure 14

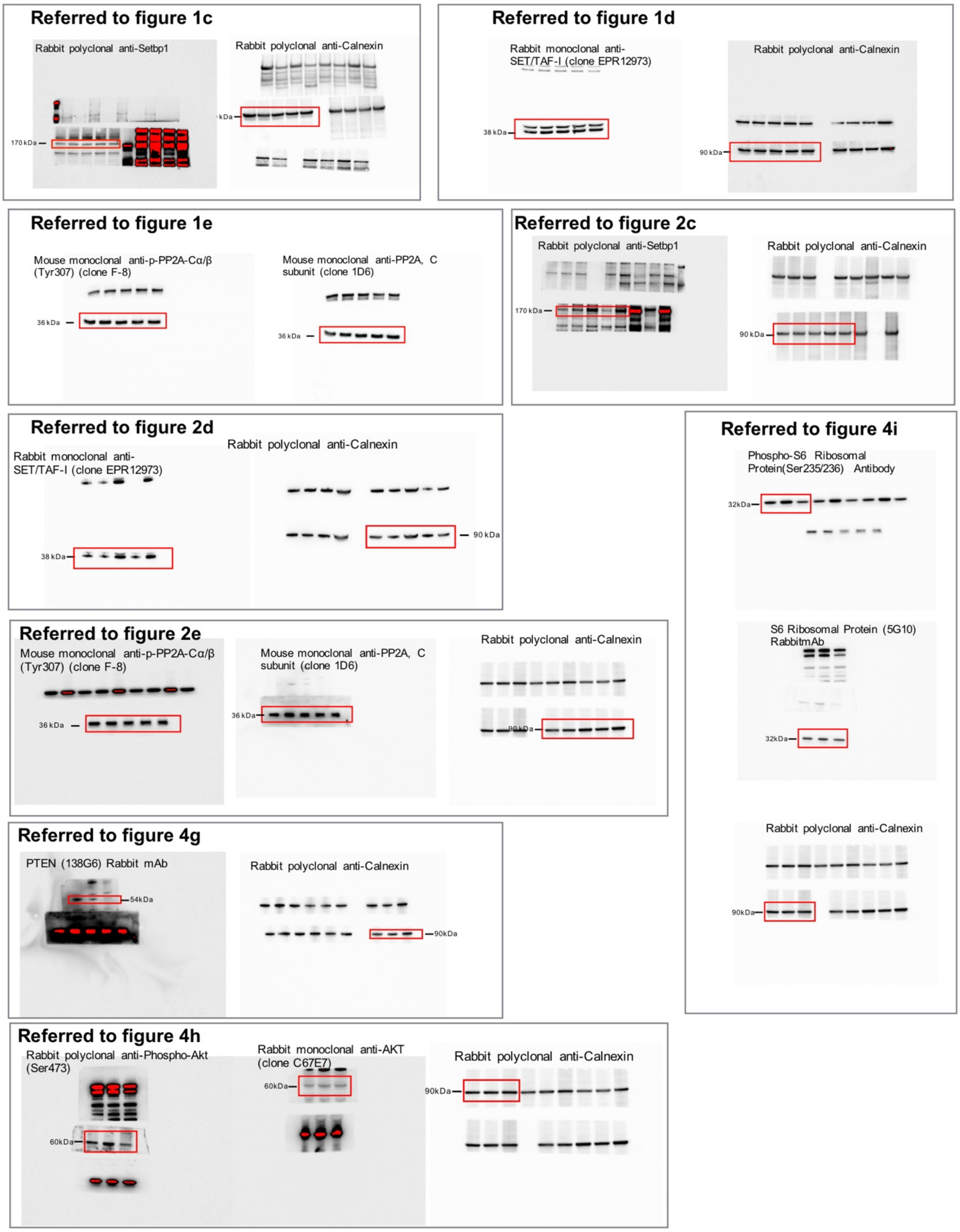

Supplementary Fig. 14 Full blots of the figures. Uncropped blots. Referred to figures 1, 2, and 4.

Supplementary Figure 15

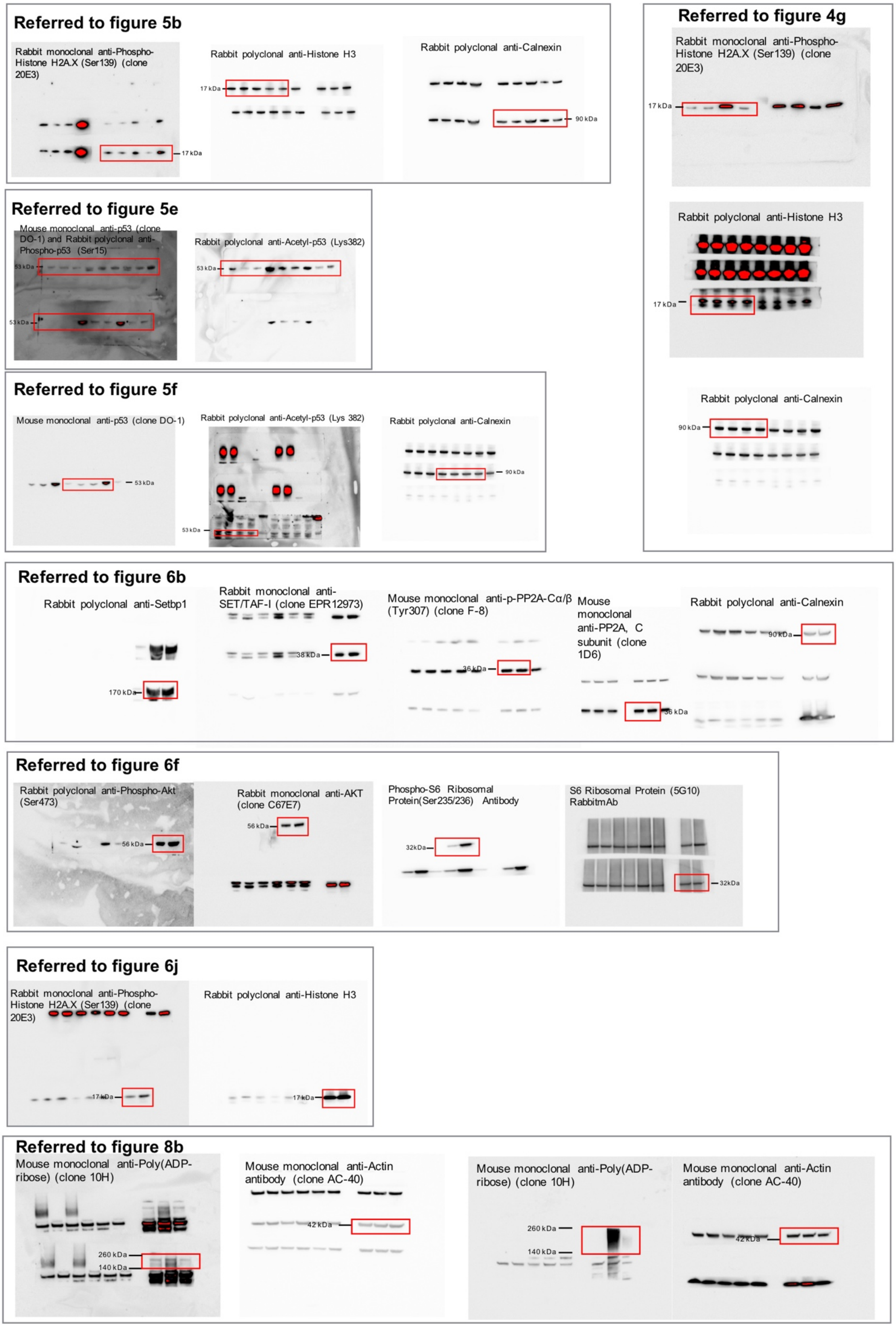

Supplementary Fig. 15 Full blots of the figures. Uncropped blots. Referred to figures 5, 6, and 8.

Supplementary Figure 16

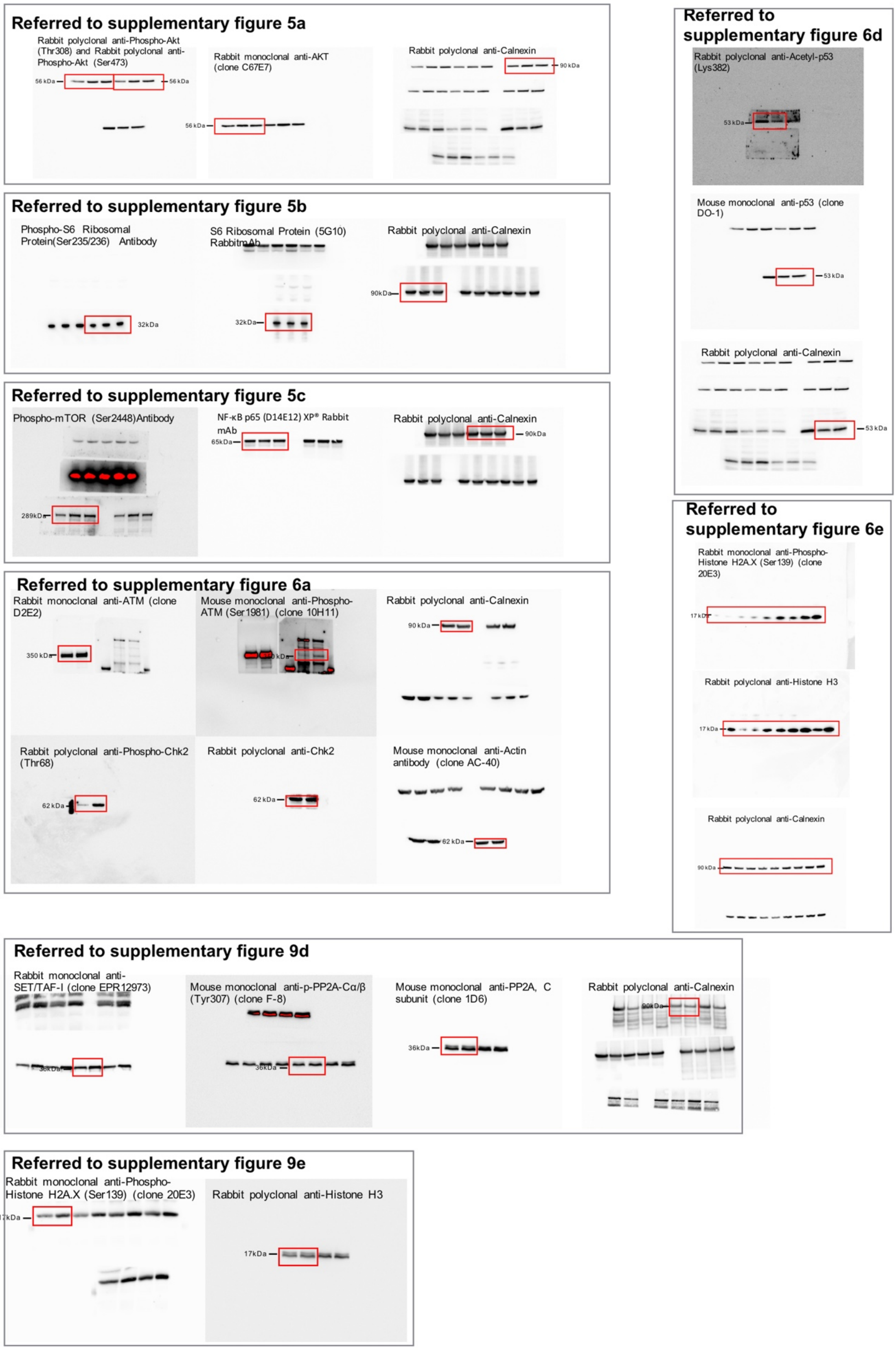

Supplementary Fig. 16 Full blots of the figures. Uncropped blots. Referred to supplementary figures 5, 6, and 9.

Supplementary Figure 17

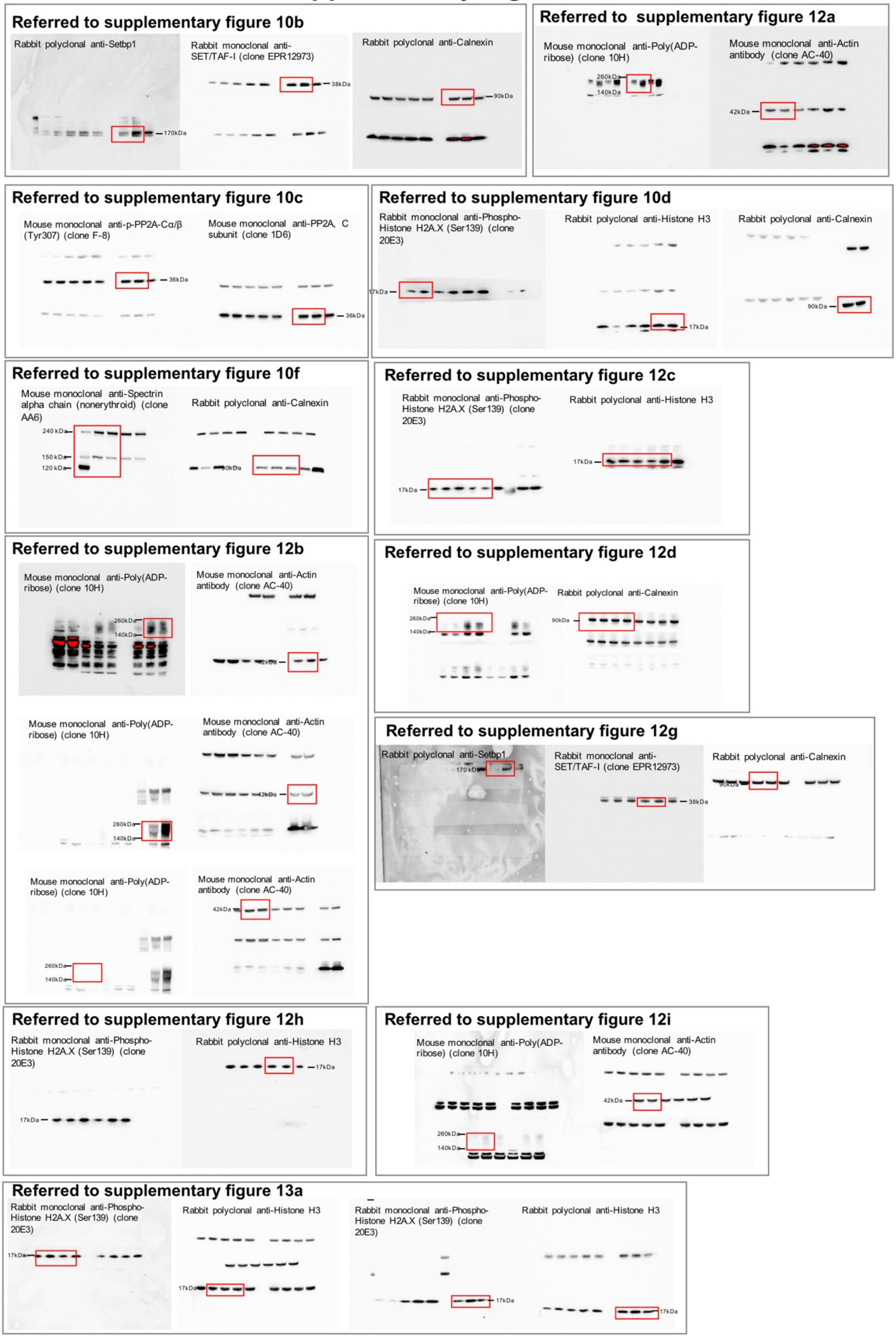

Supplementary Fig. 17 Full blots of the figures. Uncropped blots. Referred to supplementary figures 10, 12, and 13.

SUPPLEMENTARY DATASET LEGENDS

Supplementary Dataset 1. Genes deregulated in SGS neural precursor cells compared to control. Differentially expressed genes found in our NGS analyses (sheets 1, 2). Gene list used for the Heatmap in Figure S8e (sheet 3).

Supplementary Dataset 2. Gene Ontology for genes deregulated in SGS NPCs. Gene Ontology analysis of differentially expressed genes

Supplementary Dataset 3. Statistical details related to figures 1, 2, 3, 5, 8, and Supplementary Fig. 2, 6 and 7. Summary of statistical tests used for the figures: 1d (sheet 1), 2d (sheet 2), 3a (sheet 3), supplementary 2h (sheet 4), 5e (sheet 5), supplementary 6e (sheet 6), 8c (sheet 7), 8g (sheet 8), supplementary 7b (sheet 9).

Supplementary Dataset 4. List of primers. List of the oligonucleotides used in this work.

Supplementary Dataset 5. List of reagents' details List of the details of the reagents and datasets used in this work.
