# Supplementary material for: SETBP1 accumulation induces P53 inhibition and genotoxic stress in neural progenitors underlying neurodegeneration in Schinzel-Giedion syndrome
Source: Nat Commun. 2021 Jun 30;12:4050. doi: 10.1038/s41467-021-24391-3 (PMC8245514; doi:10.1038/s41467-021-24391-3)
Supplement: Supplementary file 3 — Description of Additional Supplementary Files [file 41467_2021_24391_MOESM3_ESM.docx]

Description of Additional Supplementary Files

Title: Supplementary Dataset 1.

Description: Genes deregulated in SGS neural precursor cells compared to control. Differentially expressed genes found in our NGS analyses (sheets 1, 2). Gene list used for the Heatmap in Figure S8e (sheet 3).

Title: Supplementary Dataset 2.

Description: Gene Ontology for genes deregulated in SGS NPCs. Gene Ontology analysis of differentially expressed genes

Title: Supplementary Dataset 3.

Description: Statistical details related to figures 1, 2, 3, 5, 8, and Supplementary Fig. 2, 6 and 7. Summary of statistical tests used for the figures: 1d (sheet 1), 2d (sheet 2), 3a (sheet 3), supplementary 2h (sheet 4), 5e (sheet 5), supplementary 6e (sheet 6), 8c (sheet 7), 8g (sheet 8), supplementary 7b (sheet 9).

Title: Supplementary Dataset 4.

Description: List of primers. List of the oligonucleotides used in this work.

Title: Supplementary Dataset 5.

Description: List of reagents’ details List of the details of the reagents and datasets used in this work.
