# Supplementary material for: SETBP1 accumulation induces P53 inhibition and genotoxic stress in neural progenitors underlying neurodegeneration in Schinzel-Giedion syndrome
Source: Nat Commun. 2021 Jun 30;12:4050. doi: 10.1038/s41467-021-24391-3 (PMC8245514; doi:10.1038/s41467-021-24391-3)
Supplement: Supplementary file 9 — Reporting Summary [file 41467_2021_24391_MOESM9_ESM.pdf]

nature research

Corresponding author(s): Alessandro Sessa
Last updated by author(s): Jun 10, 2021

Reporting Summary

Nature Research wishes to improve the reproducibility of the work that we publish. This form provides structure for consistency and transparency in reporting. For further information on Nature Research policies, see our Editorial Policies and the Editorial Policy Checklist.

Statistics

For all statistical analyses, confirm that the following items are present in the figure legend, table legend, main text, or Methods section.

n/a	Confirmed	
☐	☒	The exact sample size (*n*) for each experimental group/condition, given as a discrete number and unit of measurement
☐	☒	A statement on whether measurements were taken from distinct samples or whether the same sample was measured repeatedly
☐	☒	The statistical test(s) used AND whether they are one- or two-sided<br>*Only common tests should be described solely by name; describe more complex techniques in the Methods section.*
☒	☐	A description of all covariates tested
☒	☐	A description of any assumptions or corrections, such as tests of normality and adjustment for multiple comparisons
☐	☒	A full description of the statistical parameters including central tendency (e.g. means) or other basic estimates (e.g. regression coefficient) AND variation (e.g. standard deviation) or associated estimates of uncertainty (e.g. confidence intervals)
☐	☒	For null hypothesis testing, the test statistic (e.g. F, t, r) with confidence intervals, effect sizes, degrees of freedom and P value noted<br>*Give P values as exact values whenever suitable.*
☒	☐	For Bayesian analysis, information on the choice of priors and Markov chain Monte Carlo settings
☒	☐	For hierarchical and complex designs, identification of the appropriate level for tests and full reporting of outcomes
☒	☐	Estimates of effect sizes (e.g. Cohen's *d*, Pearson's *r*), indicating how they were calculated

Our web collection on statistics for biologists contains articles on many of the points above.

Software and code

Policy information about availability of computer code

Data collection	No software was used
Data analysis	Cellranger v4.0.0<br>Seurat v4.0<br>STAR aligner v2.7.9.a<br>DESeq2 v1.32<br>GSEA v4.1.0<br>GENE-E v3.0.215<br>Ikaros (v 5.8.12) (MetaSystems)<br>FCS Express 6.06.0040 (De NovoSoftware)<br>Fiji v 1.48i<br>CFX Manager Software v3.1<br>GraphPad Prism software v6.0c

For manuscripts utilizing custom algorithms or software that are central to the research but not yet described in published literature, software must be made available to editors and reviewers. We strongly encourage code deposition in a community repository (e.g. GitHub). See the Nature Research guidelines for submitting code & software for further information.

Data

Policy information about availability of data

All manuscripts must include a data availability statement. This statement should provide the following information, where applicable:

- Accession codes, unique identifiers, or web links for publicly available datasets
- A list of figures that have associated raw data
- A description of any restrictions on data availability

Genomic data are available GEO accession GSE171266, (go to https://www.ncbi.nlm.nih.gov/geo/query/acc.cgi?acc=GSE171266)

Field-specific reporting

Please select the one below that is the best fit for your research. If you are not sure, read the appropriate sections before making your selection.

- [x] Life sciences
- [ ] Behavioural & social sciences
- [ ] Ecological, evolutionary & environmental sciences

For a reference copy of the document with all sections, see nature.com/documents/nr-reporting-summary-flat.pdf

Life sciences study design

All studies must disclose on these points even when the disclosure is negative.

Sample size	No statistical methods were used to pre-determine sample sizes, but our sample sizes are similar to those reported in previous publications (see Conforti P. et al., 2020; Inak G. et al., 2021; Xie Y. et al., 2020; for experiments concerning isogenic cell line derivation, PSCs characterization and 2D differentiation, and Inak G. et al; Klaus J. et al. 2019 for experiments concerning organoids). We used independent control lines and isogenic control lines to increase the robustness of the results. However, sample size per group and condition is equal to or greater than generally accepted standard of three biological replications per group.
Data exclusions	No data were excluded
Replication	We repeated all experiments using at least three biological replicates over distinct independent experiments. We specified the number of biological replicates and independent experiments in the respective figure legends.
Randomization	We plated the cells in a random distribution onto cell culture and multi-well plate positions, and randomly assigned them to experimental groups. We perfomed cell counting on random miscroscope view fields.
Blinding	The investigator who performed RNA sequencing were blinded to the genotype. Other data collection and analyses were not performed blind to the conditions due to obvious differences between groups. The same results have been repeated by multiple members of the research team.

Reporting for specific materials, systems and methods

We require information from authors about some types of materials, experimental systems and methods used in many studies. Here, indicate whether each material, system or method listed is relevant to your study. If you are not sure if a list item applies to your research, read the appropriate section before selecting a response.

Materials & experimental systems

n/a	Involved in the study	
[ ]	[x]	Antibodies
[ ]	[x]	Eukaryotic cell lines
[x]	[ ]	Palaeontology and archaeology
[x]	[ ]	Animals and other organisms
[ ]	[x]	Human research participants
[x]	[ ]	Clinical data
[x]	[ ]	Dual use research of concern

Methods

n/a	Involved in the study	
[x]	[ ]	ChIP-seq
[ ]	[x]	Flow cytometry
[x]	[ ]	MRI-based neuroimaging

Antibodies

Antibodies used	The antibodies used in this work are listed with the details (producer, species or origin, dilution of usage, and RRID) in the Supplementary Table 2.
Validation	The antibodies used in this work are all commercial and widely tested in literature for the proposed assay.<br><br>Mouse monoclonal anti-TRA-1-60: Sigma-Aldrich, Cat# MAB4360, RRID: AB_11211864

Culture of human embryonic stem cells on human and mouse feeder cells.
Gautam Dravid, Holly Hammond, Linzhao Cheng, 2006. DOI: 10.1385/1-59745-046-4:91

Mouse monoclonal anti-SOX2 (Clone # 245610): R&D system, Cat# MAB2018, RRID:AB_358009
Generation of the human induced pluripotent stem cell (hiPSC) line PSMi006-A from a patient affected by an autosomal recessive form of long QT syndrome type 1
Manuela Mura et al., 2019. DOI: 10.1385/1-59745-046-4:91

Rabbit polyclonal anti-Oct4: Abcam, Cat#ab18976, RRID:AB_444714
Derivation of Pluripotent Stem Cells with In Vivo Embryonic and Extraembryonic Potency.
Yang Y etal., 2017. DOI: 10.1016/j.cell.2017.02.005.

Rabbit polyclonal anti-Nanog: Abcam, Cat#ab106465, RRID:AB_10858563
RNA Helicase DDX5 Inhibits Reprogramming to Pluripotency by miRNA-Based Repression of RYBP and its PRC1-Dependent and -Independent Functions
Huanhuan Li et al., 2017. DOI: 10.1016/j.stem.2016.12.002

Rabbit polyclonal anti-Tubulin β-3: Covance, Cat#PRB-435P, RRID:AB_291637
Driving Neuronal Differentiation through Reversal of an ERK1/2-miR-124-SOX9 Axis Abrogates Glioblastoma Aggressiveness
Hanna Sabelström et al., 2019. DOI: 10.1016/j.celrep.2019.07.071

Rabbit polyclonal anti-Pax6 (Clone Poly19013): Covance, Cat# PRB-278P, RRID:AB_291612
Merging organoid and organ-on-a-chip technology to generate complex multi-layer tissue models in a human retina-on-a-chip platform
Kevin Achberger et al., 2019. DOI: 10.7554/eLife.46188

Mouse monoclonal anti-Actin, α-Smooth Muscle: Sigma-Aldrich, Cat#A5228, RRID:AB_262054
Tissue-Engineered Vascular Grafts with Advanced Mechanical Strength from Human iPSCs
Jiesi Luo et al., 2020. DOI: 10.1016/j.stem.2019.12.012

Rabbit polyclonal anti-Foxa2: Abcam, Cat#ab40874, RRID:AB_732411
Cerebral dopamine neurotrophic factor is essential for enteric neuronal development, maintenance, and regulation of gastrointestinal transit
Alcmène Chalazonitis et al., 2020. DOI: 10.1002/cne.24901

Mouse monoclonal anti-Nestin (clone 10C2): Sigma-Aldrich, Cat# MAB5326, RRID:AB_2251134
Subventricular zone neural progenitors from rapid brain autopsies of elderly subjects with and without neurodegenerative disease
Brian W Leonard et al., 2009. DOI: 10.1002/cne.22040

Rabbit polyclonal anti-phospho-Histone H3 (Ser10): Sigma-Aldrich, Cat#06-570, RRID:AB_310177
Tight junction protein occludin regulates progenitor Self-Renewal and survival in developing cortex
Raphael M Bendriem et al., 2019. DOI: 10.7554/eLife.49376

Rabbit polyclonal anti-Foxg1: Abcam, Cat#ab18259, AB_732415
Mechanisms of hyperexcitability in Alzheimer's disease hiPSC-derived neurons and cerebral organoids vs isogenic controls
Swagata Ghatak et al., 2019. DOI: 10.7554/eLife.50333

Mouse monoclonal anti-ZO1 (clone ZO1-1A12), FITC: Thermo Fisher Scientific, Cat#33-9111, RRID:AB_2533148
A multiplex high-throughput gene expression assay to simultaneously detect disease and functional markers in induced pluripotent stem cell-derived retinal pigment epithelium
Marc Ferrer et al., 2014. DOI: 10.5966/sctm.2013-0192

Rabbit monoclonal anti-Phospho-Histone H2A.X (Ser139) (clone 20E3): Cell Signaling Technology, Catt#9718, RRID:AB_2118009
WB: PARP1 inhibitors trigger innate immunity via PARP1 trapping-induced DNA damage response
Chiho Kim et al., 2020. DOI: 10.7554/eLife.60637
IF: A high-throughput small molecule screen identifies farrerol as a potentiator of CRISPR/Cas9-mediated genome editing
Weina Zhang et al., 2020. DOI: 10.7554/eLife.56008

Rabbit polyclonal anti-Cleaved Catspase-3 (Asp175): Cell Signaling Technology, Cat#9661, RRID:AB_2341188
mTOR signaling regulates the morphology and migration of outer radial glia in developing human cortex
Madeline G Andrews et al., 2020. DOI: 10.7554/eLife.58737

Rabbit polyclonal anti-Catlnexin: Sigma-Aldrich, Cat#C4731, RRID:AB_476845
PI4KB on Inclusion Bodies Formed by ER Membrane Remodeling Facilitates Replication of Human Parainfluenza Virus Type 3
Zhifei Li et al., 2019. DOI: 10.1016/j.celrep.2019.10.052

Chicken polyclonal anti-GFP: Thermo Fisher Scientific, Cat#A10262, RRID:AB_2534023
Maturation of spinal motor neurons derived from human embryonic stem cells
Tomonori Takazawa et al., 2012. DOI: 10.1371/journal.pone.0040154

Chicken polyclonal anti-Tbr1: Sigma-Aldrich, Cat# AB2261, RRID:AB_10615497
Two microcephaly-associated novel missense mutations in CASK specifically disrupt the CASK-neurexin interaction
Leslie E W LaConte et al., 2018. DOI: 10.1007/s00439-018-1874-3

Chicken polyclonal anti-MAP2: Abcam, Cat#ab92434, RRID:AB_2138147
Cellular alterations identified in pluripotent stem cell-derived midbrain spheroids generated from a female patient with progressive external ophthalmoplegia and parkinsonism who carries a novel variation (p.Q811R) in the POLG1 gene
Margarita Chumarina et al., 2019. DOI: 10.1186/s40478-019-0863-7

Rabbit monoclonal anti-SET/TAF-I (clone EPR12973): Abcam, Cat#ab181990, RRID:AB_2737445
A Membraneless Organelle Associated with the Endoplasmic Reticulum Enables 3'UTR-Mediated Protein-Protein Interactions
Weirui Ma and Christine Mayr, 2018. DOI: 10.1016/j.cell.2018.10.007

Rabbit polyclonal anti-Setbp1: Proteintech, Cat#16841-1-AP, RRID:AB_2185750
Somatic SETBP1 mutations in myeloid malignancies
Hideki Makishima et al., 2013. DOI: 10.1038/ng.2696

Mouse monoclonal anti-PP2A, C subunit (clone 1D6): Sigma-Aldrich, Cat#05-421, RRID:AB_309726
Recurrent SETBP1 mutations in atypical chronic myeloid leukemia
Rocco Piazza et al., 2013. DOI: 10.1038/ng.2495

Rabbit monoclonal anti-Ki-67 (Clone SP6): ImmunologiCatl Sciences, Cat# MAB-90948
http://www.immunologicalsciences.com/p134868/KI-67-MAB-90948

Mouse monoclonal anti-p-PP2A-Cα/β (Tyr307) (clone F-8): Santa Cruz Biotechnolgy, Cat#sc-271903, RRID:AB_10611810
Hypoxia modulates protein phosphatase 2A through HIF-1α dependent and independent mechanisms in human aortic smooth muscle cells and ventricular cardiomyocytes
Ismail Suliman Elgenaidi and James Paul Spiers, 2019. DOI: 10.1111/bph.14648

Rabbit monoclonal anti-AKT (clone C67E7): Cell Signaling Technology, Cat#4691, RRID:AB_915783
The peripheral CB 1 receptor antagonist JD5037 attenuates liver fibrosis via a CB 1 receptor/β-arrestin1/Akt pathway
Siwei Tan et al., 2020. DOI: 10.1111/bph.15010

Rabbit polyclonal anti-Phospho-Akt (Ser473): Cell Signaling Technology, Cat#9271, RRID:AB_329825
Three-dimensional growth of breast cancer cells potentiates the anti-tumor effects of unacylated ghrelin and AZP-531
CheukMan C Au et al-, 2020. DOI: 10.7554/eLife.56913

Rabbit polyclonal anti-Phospho-Akt (Thr308): Cell Signaling Technology, Cat#9275, RRID:AB_329828
Trimethylamine N-Oxide Binds and Activates PERK to Promote Metabolic Dysfunction
Sifan Chen et al., 2019. DOI: 10.1016/j.cmet.2019.08.021

Rabbit polyclonal anti-Histone H3: Abcam, Cat# ab1791, RRID:AB_302613
The integrated stress response induces R-loops and hinders replication fork progression
Josephine Ann Mun Yee Choo et al., 2020. DOI: 10.1038/s41419-020-2727-2

Mouse monoclonal anti-Actin antibody (clone AC-40): Sigma-Aldrich, Cat#A3853, RRID:AB_262137
SETD2 mutation in renal clear cell carcinoma suppress autophagy via regulation of ATG12
Patricia González-Rodríguez et al., 2020. DOI: 10.1038/s41419-020-2266-x

Rabbit monoclonal anti-ATM (clone D2E2): Cell Signaling Technology, Cat#2873, RRID:AB_2062659
FBXW7 Confers Radiation Survival by Targeting p53 for Degradation
Danrui Cui et al., 2020. DOI: 10.1016/j.celrep.2019.12.032

Mouse monoclonal anti-Phospho-ATM (Ser1981) (clone 10H11): Thermo Fisher Scientific, Cat#MA1-2020, RRID:AB_1086244
2-Hydroxyethyl methacrylate-induced apoptosis through the ATM- and p53-dependent intrinsic mitochondrial pathway
Helmut Schweikl et al., 2014. DOI: 10.1016/j.biomaterials.2013.12.044

Rabbit polyclonal anti-Chk2: Cell Signaling Technology, Cat#2662, RRID:AB_2080793
Insulin Signaling Regulates the FoxM1/PLK1/CENP-A Pathway to Promote Adaptive Pancreatic β Cell Proliferation
Jun Shirakawa et al., 2017. DOI: 10.1016/j.cmet.2017.02.004

Rabbit polyclonal anti-Phospho-Chk2 (Thr68): Cell Signaling Technology, Cat#2661, RRID:AB_331479
Torin2 Exploits Replication and Checkpoint Vulnerabilities to Cause Death of PI3K-Activated Triple-Negative Breast Cancer Cells
Sameer S Chopra et al., 2020. DOI: 10.1016/j.cels.2019.11.001

Mouse monoclonal anti-p53 (clone DO-1): Santa Cruz Biotechnolgy, Cat#sc-126, RRID:AB_628082
Metformin inhibits androgen-induced IGF-IR up-regulation in prostate cancer cells by disrupting membrane-initiated androgen signaling

Roberta Malaguarnera et al., 2014. DOI: 10.1210/en.2013-1925

Rabbit polyclonal anti-Acetyl-p53 (Lys382): Cell Signaling Technology, Cat#2525, RRID:AB_330083
SIRT1 Activation Disrupts Maintenance of Myelodysplastic Syndrome Stem and Progenitor Cells by Restoring TET2 Function
Jie Sun et al., 2018. DOI: 10.1016/j.stem.2018.07.018

Rabbit polyclonal anti-Phospho-p53 (Ser15): Cell Signaling Technology, Cat#9284, RRID:AB_331464
p53 Loss in Breast Cancer Leads to Myc Activation, Increased Cell Plasticity, and Expression of a Mitotic Signature with Prognostic Value
Angela Santoro et al., 2019. DOI: 10.1016/j.celrep.2018.12.071

Mouse monoclonal anti-Spectrin alpha chain (nonerythroid) (clone AA6): Millipore, Cat#MAB1622, RRID:AB_11214057
Neuronally Enriched RUFY3 Is Required for Caspase-Mediated Axon Degeneration
Nicholas T Hertz et al., 2019. DOI: 10.1016/j.neuron.2019.05.030

Mouse monoclonal anti-Poly(ADP-ribose) (clone 10H): Enzo Life Sciences, Cat#ALX-804-220-R100, RRID:AB_2052275
Olaparib-induced Adaptive Response Is Disrupted by FOXM1 Targeting that Enhances Sensitivity to PARP Inhibition
Pingping Fang et al., 2018. DOI: 10.1158/1541-7786.MCR-17-0607

PTEN (138G6) Rabbit mAb: Cell Signaling Technology, Cat#9559, RRID:AB_390810
Replication Study: A coding-independent function of gene and pseudogene mRNAs regulates tumour biology
John Kerwin et al., 2020. DOI: 10.7554/eLife.51019

Rabbit phospho-S6 Ribosomal Protein (Ser235/236) Antibody: Cell Signaling Technology, Cat#2211, RRID:AB_331679
Dysregulation of Mitochondrial Ca 2+ Uptake and Sarcolemma Repair Underlie Muscle Weakness and Wasting in Patients and Mice Lacking MICU1
Valentina Debattisti et al., 2019. DOI: 10.1016/j.celrep.2019.09.063

S6 Ribosomal Protein (5G10) Rabbit mAb: Cell Signaling Technology, Cat#2217, RRID:AB_331355
Dysregulation of Mitochondrial Ca 2+ Uptake and Sarcolemma Repair Underlie Muscle Weakness and Wasting in Patients and Mice Lacking MICU1
Valentina Debattisti et al., 2019. DOI: 10.1016/j.celrep.2019.09.063

Rabbit phospho-mTOR (Ser2448) Antibody: Cell Signaling Technology, Cat#2971, RRID:AB_330970
CD74 knockout protects against LPS-induced myocardial contractile dysfunction through AMPK-Skp2-SUV39H1-mediated demethylation of BCLB
Yuanfei Luo et al., 2020. DOI: 10.1111/bph.14959

Rabbit anti-GABA antibody: Sigma-Aldrich, Cat#A2052, RRID:AB_477652
The Role of Sonic Hedgehog in the Specification of Human Cortical Progenitors In Vitro
Nevena V Radonjić et al., 2016. DOI: 10.1093/cercor/bhu183

Chicken polyclonal anti-Vimentin antibody: Abcam, Cat#ab24525, RRID:AB_778824
Macrophage-Derived Slit3 Controls Cell Migration and Axon Pathfinding in the Peripheral Nerve Bridge
Xin-Peng Dun et al., 2019. DOI: 10.1016/j.celrep.2018.12.081

Anti-Doublecortin antibody: Abcam, Cat#ab18723, RRID: AB_732011
In vitro human stem cell derived cultures to monitor calcium signaling in neuronal development and function
Yojet Sharma et al., 2020. DOI: 10.12688/wellcomeopenres.15626.1

NF-κB p65 (D14E12) XP® Rabbit mAb: Cell Signaling Technology, Cat#8242, RRID:AB_10859369
IL-1β Inhibits Connexin 43 and Disrupts Decidualization of Human Endometrial Stromal Cells Through ERK1/2 and p38 MAP Kinase
Jie Yu et al., 2017. DOI: 10.1210/en.2017-00495.

Eukaryotic cell lines

Policy information about cell lines

Cell line source(s)	Patients and control fibroblasts were gift from Dr. Alexander Hoischen (Radboud umc, the Nederlands). We derived IPSCs from these fibroblasts.<br><br>HEK293T: ATCC, Cat# CRL-3216.
Authentication	Fibroblasts were tested for mycoplasma, Karyotype and for the presence of expected mutations (Sanger sequencing) before their usage for reprogramming. Derived IPSCs were routinely tested for mycoplasma, stemness and pluripotency, Karyotype and for the presence of expected mutations (Sanger sequencing).

Mycoplasma contamination	all cell lines were negative for mycoplama contamination (PCR-test)
Commonly misidentified lines (See ICLAC register)	No commonly misidentified cell lines were used.

Human research participants

Policy information about studies involving human research participants

Population characteristics	Fibroblasts obtained from two SGS kids (see Hoischen et al., Nat Gen, 2010). No need of particular information since it is not a covariate-relevant population
Recruitment	Schinzel-Giedion syndrome patients fulfilled the diagnostic criteria suggested by (Lehman et al. Am. J. Med. Genet., 2008) and are from various parts of the world (Europe (n=7), New- Zealand (n=3), Australia (n=2) and the USA (n=1)) (Hoischen et al., Nat Gen, 2010). We obtained from Hoischen and colls cells of two of them. Since the rarity of the disease we do not perform statistical calculation on the patients population thus no biases (including self-selection bias) are applied here.
Ethics oversight	Medical Ethics Committee of the Radboud University Nijmegen Medical Center, Nijmegen, The Netherlands.

Note that full information on the approval of the study protocol must also be provided in the manuscript.

Flow Cytometry

Plots

Confirm that:

- [x] The axis labels state the marker and fluorochrome used (e.g. CD4-FITC).
- [x] The axis scales are clearly visible. Include numbers along axes only for bottom left plot of group (a 'group' is an analysis of identical markers).
- [ ] All plots are contour plots with outliers or pseudocolor plots.
- [x] A numerical value for number of cells or percentage (with statistics) is provided.

Methodology

Sample preparation	Neural progenitor cells diffrentiated from human iPSCs were seed on matrigel-coated plates at a density of 80000 cells/cm2. 24 hours after seeding, cells were detached with Accutase solution, harvested and fixed in 70% ethanol on ice for 2 hours; after washing with PBS, fixed cell were treated with RNase A (200 µg/ml, Carlo Erba) and stained with propidium iodide (50 µg/ml, ThermoFisher Scientific) at 37ºC in the dark for 20 minutes. Samples were immediately acquired on FACS Canto II (BD) flow cytometer and cell cycle profiles were analyzed using FCS Express 6 Flow (De NovoSoftware) and expressed as percentage. In order to analyze G1/S exit, cells were synchronized by double thymidine block and analyzed by FACS at different time points after thymidine release. Briefly, 24 hours after seeding thymidine (2mM, Sigma-Aldrich) was added to culture medium and cells were cultured for 18 hours; then thymidine was removed and cells were released for 9 hours in fresh NPC medium. A second round of thymidine (2mM) was added for 18 hours. Then cells were released by washing with 1x PBS and incubating them in fresh NPC medium. Cells were collected at 0, 2, 10 and 24 hours after release for propidium iodide staining and FACS analysis as described before.
Instrument	FACS Canto II (BD) flow cytometer
Software	FCS Express 6 Flow (De NovoSoftware)
Cell population abundance	Abundance of cell population in each phase of cell cycle is described as percentage on total number of cells based on P2 gate. See the bar graph in figure 3c, supplementary figures 2g and 6b for details of cell percentage in each phase of cell cycle, and supplementary figure 3 for gating strategy.
Gating strategy	FSC-A/FSC-H were used to exclude doublets and FSC-A/SSC-A were used to exclude debris. Cell count were identified on P2 gate by propidium iodide staining and gates that identify different cell cycle phases were set according to peak distribution (P3: apoptotic cells; P4: G1-phase; P5: S-phase; P6: M/G2-phase; P7: cells with DNA content > 4N). See Supplementary figure 3.

- [x] Tick this box to confirm that a figure exemplifying the gating strategy is provided in the Supplementary Information.
